# *Chlamydia trachomatis* serovar D replicates in uroepithelial T24/83 cells in the absence of overt inflammation

**DOI:** 10.1038/s41598-026-62091-4

**Published:** 2026-07-20

**Authors:** Simone Albrecht, Svetlana Kuhn, Lina Kellner, Xaver Rait, Isabelle Müller, Leon Heine, Hannah Griffiths, Carolina de la Torre, Norbert Gretz, Thomas Miethke

**Affiliations:** 1 Institute of Medical Microbiology and Hygiene, Medical Faculty Mannheim, Heidelberg University, Theodor-Kutzer-Ufer 1-3, 68167 Mannheim, Germany; 2 Mannheim Institute for Innate Immunoscience (MI3), Franz-Volhard-Str. 6, 68167 Mannheim, Germany; 3 NGS Core Facility, Medical Faculty Mannheim, Heidelberg University, Theodor-Kutzer-Ufer 1-3, 68167 Mannheim, Germany; 4 Medical research center, Medical Faculty Mannheim, Heidelberg University, Theodor-Kutzer-Ufer 1-3, 68167 Mannheim, Germany

**Keywords:** *Chlamydia trachomatis*, Epithelial cells, Inflammation, Infectivity, Toll-like receptor 3 and 4, Diseases, Immunology, Microbiology, Pathogenesis

## Abstract

The human uroepithelial bladder cancer cell line T24/83 replicates *C. trachomatis* serovar D efficiently. We show here that the infection with the pathogen induces significant transcription of cytokine genes such as *il1β*, *il6* and *tnf* but not their secretion. Infected HeLa cells also do not release these cytokines with the exception of IL-6. In contrast, endotoxin, transfected polyI: C or infection with the uropathogenic *E. coli* strain CFT073 induce a robust pro-inflammatory response in T24/83 cells. These findings indicate that the cells recognize pathogen-associated molecular patterns including the TLR4 and TLR3 ligands endotoxin and dsRNA, respectively, also present in *C. trachomatis* serovar D. We, therefore, generated *tlr4-*deficient T24/83 cells to study its influence on replication of *C. trachomatis* serovar D. In contrast to our expectation, *C. trachomatis* serovar D replication in T24/83 cells did not benefit from *tlr4-*deficiency. TLR3 was reported to recognize *C. trachomatis* serovar D in the human oviduct cell line OE-E6/E7. However, *tlr3-*deficiency also did not improve the replication of the pathogen in T24/83 cells. Interestingly, *C. trachomatis* serovar D did not modulate an endotoxin-induced cytokine release but impaired significantly a polyI: C-induced and TLR3-dependent release of IFN-β. We conclude that *C. trachomatis* serovar D avoids an inflammatory reaction and circumvents recognition by TLR4 and TLR3 in infected uroepithelial T24/83 cells.

**Supplementary Information:**

The online version contains supplementary material available at 10.1038/s41598-026-62091-4.

## Introduction

Infection of females with *Chlamydia trachomatis* serovars D-K may lead to hydrosalpinx, extra-uterine gravidity, infertility or pelvic inflammatory disease presumably due to long lasting inflammatory responses of cells of the urogenital tract. However, many cases are symptomless. The obligate intracellular bacterium is sexually transmitted and responsible for human genital tract infections, which now reached the number of 131 million cases per year^1,2^. Epithelial cells of the urethra or the genital tract are the prime target of infection with *C. trachomatis* serovars D-K and support the unique chlamydial replication cycle without restriction in contrast to other cells such as macrophages or polymorphonuclear neutrophils^3^. Other epithelial cells like bronchial or bladder epithelial cells also replicate the microorganisms efficiently. The replication cycle includes elementary bodies (EBs), which are metabolically inactive but infectious. EBs enter epithelial cells and reside in the cytosol within a membrane-bound vacuole called an inclusion. EBs convert to reticulate bodies (RBs), which are metabolically active and replicate, but are noninfectious. Forty to forty-eight hours post infection RBs revert to EBs after at least six rounds of replication and reduction in size, which subsequently leave the host cells to infect neighboring ones^4^.

An infection with *C. trachomatis* serovars D-K will cause symptoms, if it triggers inflammation. Inflammation, in turn, should be initiated in cells where *C. trachomatis* serovars D-K replicate, i.e. in epithelial cells. Accordingly, human epithelial cells of the urogenital tract express pattern recognition receptors (PRRs) such as Toll-like receptors (TLRs) and their key adaptor molecule myeloid factor of differentiation 88 (MyD88), but also other PRRs such as cyclic GMP-AMP synthase/Stimulator of Interferon Genes (cGAS/STING)^5–10^. It appears that chlamydial inclusions attract transfected TLR2 and MyD88 as well as endogenous cGAS/STING in epithelial cells indicating they are involved in the intracellular recognition of the pathogen^7,9^. In addition, TLR3 mediates cytokine secretion of the *C. trachomatis* serovar D infected human oviduct cell line OE-E6/E7 and controls the size of chlamydial inclusions in these cells^11^. Thus, epithelial cells express PRRs required to initiate inflammation.

To further address this issue, Tang et al. analyzed *C. trachomatis* serovar L2 or D infected primary human ectocervical cells and demonstrated that both serovars induced the transcription of the cytokines/chemokine IL-6, IL-8, TNF-α and CCL5^12^. However, they did not investigate whether these cytokines/chemokine transcripts were also translated and detectable in the culture supernatant. Several studies demonstrated that infection of human epithelial cells or cervical carcinoma cell lines with *C. trachomatis* induced the transcription of several pro-inflammatory cytokine genes^13–15^. However, many of them used *C. trachomatis* L2, which is more aggressive in vivo and in vitro than *C. trachomatis* serovars D-K and induced higher cytokine levels compared to *C. trachomatis* serovar E^15^. In addition, some studies used prolonged incubation times of 72 h, which exceeded the chlamydial replication cycle. Thus, infected and lysed host cells may release danger signals, which could trigger the remaining host cells to secrete pro-inflammatory cytokines as was described recently in an elegant murine genital tract infection model with *C. trachomatis*^3^. This study demonstrated that chlamydial infection of neutrophils and epithelial cells caused the release of ATP, which serves as damage-associated molecular pattern (DAMP) to trigger P2X7-dependently the NLRP3 inflammasome of macrophages^3^. Interestingly, the infection with *C. trachomatis* serovar D of human polarized, immortalized, endocervical epithelial cells induced only a modest cytokine response, where levels of IL-6, TNF-α and CXCL8 were comparable to mock-infected cells^16^. In contrast, infected cells produced significantly higher IL-1α levels; however, this response required 72 h of culture^16^.

Based on these findings we hypothesize in this study, that *C. trachomatis* serovar D may multiply in cells, which allow efficient replication, without initiation of inflammation. We used the human bladder cancer cell line T24/83 for our analysis of the pro-inflammatory response upon infection with *C. trachomatis* serovar D, since it efficiently replicates the bacterium and it expresses several PRRs such as TLR4, TLR3, MyD88, cytosolic helicases and cGAS/STING. Moreover, it responds to bacterial PAMPs and to an infection with the uropathogenic *E. coli* strain CFT073 with a vigorous secretion of pro-inflammatory cytokines. Although bladder epithelial cells are not the prime target of *C. trachomatis*, chlamydial antigens were detected immuno-histologically in bladder epithelial cells in association with cystitis in 12 of 36 patients^17^. Moreover, in a murine model analyzing the infection of the genitourinary tract by *C. muridarum* in male mice post inoculation of the meatus urethra, the bladder was frequently infected^18^. Thus, bladder epithelial cells possibly harbor *C. trachomatis* during a urogenital infection.

## Materials and methods

### Cell culture

HeLa cells (Cytion, CLS 300194) were cultured in DMEM (Gibco, 41965062). The medium was supplemented with 10% FCS (Gibco, A5670701), 2 mM L-glutamine (Sigma-Aldrich/Merck, G7513-20ML), 5 mg gentamicin (Sigma-Aldrich/Merck G1272-10 ml) and 25 mg vancomycin (Sigma-Aldrich/Merck 861987-1G) per 500 ml medium. We cultured T24/83 cells (donated by Dr. P. Erben, University Medicine Mannheim) in McCoy’s 5 A (modified) medium (Gibco, 16600082), which was supplemented with 10% FCS, 5 mg gentamicin and 25 mg vancomycin per 500 ml medium. All cell lines were washed with PBS (Sigma-Aldrich/Merck, D8537), dissociated from the cell culture flask with a Trypsin-EDTA solution (Sigma-Aldrich/Merck, T4174-100ML), 10-fold diluted with PBS, resuspended in fresh medium and seeded into new flasks twice or thrice a week. We cultured all cell lines at 37 °C and 5% CO_2_.

### Propagation of bacterial pathogens

*C. trachomatis* serovar D (DSMZ, DSM-19411) was grown in HeLa-cell monolayers in high-glucose DMEM supplemented with 10% FCS, 2 mM L-glutamine, 5 mg gentamicin and 25 mg vancomycin per 500 ml medium and 1 µg/ml cycloheximide (Sigma-Aldrich/Merck, C7698-1G). *C. trachomatis* serovar D infected cells were cultivated for 48 h at 37 °C and 5% CO_2_, and the chlamydial amount was expanded by two subsequent rounds of infection. Thereafter cells were harvested and chlamydial elementary bodies were concentrated in SPG buffer (75 g Sucrose, 0.52 g KH_2_PO_4_, 1.22 g Na_2_HPO_4_, 0.72 g L-glutamic acid in 1 l H_2_O, pH 7.4) by centrifugation (22.000 x g, 4 °C, 1 h). The chlamydial elementary bodies were stored at -80 °C in sterile filtered SPG buffer. *C. trachomatis* serovar D aliquots were thawed immediately before use. We determined the number of inclusion forming units (IFU) in our chlamydial stocks using 150,000 HeLa cells seeded in 12-well plate on coverslips and incubated at 37 °C, 5% CO_2_ overnight. Cells were infected in a serial dilution of 1:100, 1:200, 1:500, 1:1000 and 1:2000 in duplicates in medium lacking FCS. After 2 h the medium was replaced with medium containing 10% FCS. 24 h p.i. cells were fixed and permeabilized with a methanol: aceton 1:1 mixture and stained with 15 µl undiluted anti-Chlamydia Multiepitope Cocktail FITC Conjugate (Progen, discontinued) on a parafilm strip in a wet chamber. The multiepitope cocktail detects EBs, RBs, intermediate forms, chlamydial inclusions and specific cell-associated antigens directly in samples. The conjugate is used for direct immunofluorescence staining combining three specific monoclonal antibodies (mab) conjugated to fluorescein isothiocyanate (FITC). One mab is specific for the genus-specific epitope located on the Chlamydia LPS and identifies all the 15 known serovars of *C. trachomatis* as well as *C. psittaci* and *C. pneumoniae*. A second mab reacts with an epitope on the Chlamydia-outer membrane complex protein (60 kDa). The third mab identifies a species-specific epitope on the major outer membrane protein (40 kDa). The combination of these 3 specific mabs provides uniform and intense staining of all the stages in the developmental cycle of the known *C. trachomatis* serovars (A-C, D-K, L1-L3), *C. psittaci* and *C. pneumoniae* strains. In 5 fields of view per coverslip the inclusions were counted, their mean and subsequently the IFU number per µl Chlamydia suspension was calculated.

The *Escherichia coli* strain CFT073 (ATCC, 700928) was grown overnight in lysogeny broth (LB, 37 °C, shaking 200 rpm). Overnight cultures were washed three times in McCoy’s 5 A medium, 5% FCS and then used to infect wild type, *tlr4-* or *tlr3-*deficient T24/83 cells at different multiplicities of infection (MOI).

### Fluorescence microscopy

Pictures from the anti-Chlamydia Multiepitope Cocktail including the Evan´s blue counterstain were taken with a Zeiss Axio Observer microscope including the camera Axiocam 702 mono and the imaging software Zen.

### Lysate preparation, cellular fractionation, SDS-PAGE and Western blot

To explore the depletion of cytosolic IκB and the phosphorylation of IRF3, we infected T24/83 and HeLa cells with *C. trachomatis* serovar D (MOI 2) in the absence of FCS and antibiotics. After 2 h, the medium was exchanged to medium containing FCS but no antibiotics and incubation continued for another 2 h at 37 °C and 5% CO_2_. Uninfected cells treated in the same manner for 4 h were used as negative controls. As controls for cytosolic IκB degradation, T24/83 and HeLa cells were stimulated with either 1 µg/ml LPS (Sigma-Aldrich/Merck L2018-5MG) or 10 ng/ml TNF-α (Thermo Fisher PHC3015) for 30 min, respectively. As a positive control for IRF3 phosphorylation of the serines 386 and 396, we transfected T24/83 and HeLa cells with 5 µg/ml polyI: C (Sigma-Aldrich/Merck 528906-10MG) for 4 h with Lipofectamine 3000 (Thermo Fisher, L3000015). To test, if Lipofectamine 3000 alone leads to IRF3 phosphorylation, we incubated T24/83 and HeLa for 4 h only with Lipofectamine 3000 as an additional negative control.

To prepare the cytosolic fraction, cells were lysed with Nonidet buffer (50 mM HEPES, pH 7.6, 120 mM NaCl, 20 mM β-glycerolphosphat, 1 mM NaF, 1 mM EDTA, pH 8.0, 1 mM EGTA, pH 7.6, 1% Nonidet P-40 (= IGEPAL CA630), 0.4 mM phenylmethylsulfonyl fluoride (PMSF), 1 mM Na_3_VO_4_, 4.3 mM n-octyl-β-D-glucopyranoside, 10% glycerol). Per 10 ml Nonidet buffer, we added one tablet protease inhibitor Complete Mini from Roche (#11836153001) and the cellular debris containing the nuclear fraction was spun down (13.000 x g, 20 min, 4 °C). The cytosolic fraction was mixed with 5x Laemmli buffer (7.2 mM bromphenol blue, 239.8 mM glycerin, 9.9 mM SDS, 0.5 M Tris-HCl, pH 6.8, 0.179 mM β-mercaptoethanol) and heated for 5 min at 95 °C. The pellet was mixed with 5x Laemmli buffer and heated for 10 min at 95 °C to prepare a nuclear lysate.

The lysates were separated by SDS-PAGE and blotted on a nitrocellulose membrane. We blocked the membrane either in 5% milk or in 5% BSA in TBS-T. This depended on the primary antibodies used against human IκB (diluted 1:2500), vinculin (diluted 1:5000), histone H3 (diluted 1:5000) and phospho-IRF3Ser386 (diluted 1:1000, all antibodies were purchased from abcam (ab32158, ab129002, ab1791 and ab76493), IRF3 (diluted 1:1000), phospho-IRF3Ser396 (diluted 1:1000) and TBK1 (diluted 1:1000, all antibodies were purchased from Cell Signaling Technology, #4302, #29047 and #3504). The primary antibodies were incubated over night at 4 °C. Secondary HRP-antibody swine-anti-rabbit (diluted 1:3000) was purchased from Dako (#P0399). The blots were incubated with WesternBright chemiluminescence substrate Quantum (Biozym, #541015) and the light flashes were detected with an ECL ChemoCam Imager HR6.0 (Intas Science Imaging) and quantified using Fiji.

### ELISAs

ELISA DUOset kits against human IL-1β, IL-6, IL-8, TNF-α and IFN-β were purchased from RnD systems (DY201, DY206, DY208, DY210, DY814 and ancillary reagent kit DY008) and used according to the manufacturer´s protocol. Color changes were detected and quantified using a TECAN Spark 10 M plate reader and the software Magellan.

To test the cytokine release upon chlamydial infection, T24/83 and HeLa cells were incubated for 2 h with *C. trachomatis* serovar D (MOI 5) in the absence of FCS and antibiotics. After 2 h, the medium was changed to medium containing FCS but no antibiotics and incubated for further 22 h at 37 °C and 5% CO_2_. Uninfected cells treated in the same manner for 24 h were used as negative controls. For positive controls, T24/83 and HeLa cells were either stimulated with 1 µg/ml LPS for 5–6 h or transfected with 5 µg/ml polyI: C for 4–6 h. We tested the cells for release of human IL-1β, IL-6, IL-8, TNF-α and IFN-β.

To test if *C. trachomatis* serovar D is able to manipulate cytokine production induced by LPS or lipofectamine-transfected polyI: C, we infected T24/83 cells under FCS- and antibiotics-free conditions for 2 h with *C. trachomatis* serovar D (MOI 5). Subsequently, we changed the medium to FCS-containing but antibiotics-free medium and incubated the infected cells for further 20 h. After that, T24/83 cells were either stimulated with 1 µg/ml LPS for 5–6 h or transfected with 5 µg/ml polyI: C for 5–6 h or only continued the incubation for 5–6 h. As negative controls, we used uninfected cells treated with FCS- and antibiotics-free medium for 2 h, and subsequently, with FCS-containing but antibiotics-free medium for 26 h. We tested the cells for production of human IL-6, IL-8 and IFN-β. We also transfected *C. trachomatis* serovar D-infected (MOI 5) T24/83 cells with dotap (Carl Roth L787.2) and polyI: C. In this case cells were infected for 2 h, subsequently stimulated with IFN-γ (Bio-Techne, 285-IF-100/CF, 200 ng/ml) for 18 h and then transfected with dotap and polyI: C (5 µg/ml).

### RNA preparation

T24/83 cells were infected with *C. trachomatis* serovar D (MOI 3 and 5) for 4–24 h in medium without FCS. After 2 h, the medium was changed to medium containing FCS and the cells were incubated for further 2–22 h. Uninfected T24/83 cells were treated in the same manner for 4–24 h and used as negative controls.

RNA was prepared by using the Qiagen RNeasy kit (#74104) with an additional DNase digestion step according to the manufacturer´s protocol.

### Reverse transcription real time PCR

1 µg of isolated RNA was used to generate cDNA with RevertAid First Strand cDNA Synthesis Kit (Thermo Scientific, K1621) using random hexamer primers. For the qRT-PCR samples were diluted 1:5 with H_2_O and analyzed using PowerUp™ SYBR™ Green Master Mix (Applied Biosystems™, A25742) on a CFX96 Deep Well C1000 Thermal Cycler (Bio-Rad). qPCR Primers were designed with the help of the online software tool from IDT (https://eu.idtdna.com/pages/tools/primerquest?returnurl=%2FPrimerquest%2FHome%2FIndex). To determine the primer efficiency (PE) a dilution series of the cDNA from a condition, which was expected to have the lowest Ct value, was prepared. The Ct values were plotted against the log-transformed concentration of the samples. A regression line and the respective equation were added to the plot in MSExcel. The slope of the regression line was used to determine primer efficiency: PE = 10^(− 1/slope)^. The expression of the RNA within a sample was calculated: Expression = 1/PE^Ct^. The mean of three technical replicates was then divided by the mean value of the house keeping gene GAPDH in the same sample for normalization. To obtain x-fold changes we divided the normalized values by the wild type control value. cDNA samples without reverse transcriptase were used as negative controls.

### Microarrays

Gene expression profiling was performed using human HuGene-2_0-st-type arrays (#902499, Thermo Fisher Scientific, Waltham, MA, USA). We tested each culture condition (i.e. control; *C. trachomatis* infected; LPS stimulated; polyI:C/lipofectamine transfected) in triplicate and each triplicate member was hybridized to an individual microarray. We run each culture condition for 4 and 24 h. We performed the different culture conditions, RNA preparation and hybridization at the same day. Biotinylated antisense cDNA was prepared according to the standard labelling protocol with the GeneChip^®^ WT Plus Reagent Kit and the GeneChip^®^ Hybridization, Wash and Stain Kit (both from Thermo Fisher Scientific). Afterwards, the hybridization on the chip was performed in a GeneChip Hybridization oven 640, then dyed in the GeneChip Fluidics Station 450 and thereafter scanned with a GeneChip Scanner 3000. With the exception of the control condition, where one of three chips failed to hybridize, we obtained three independent transcriptomes of each condition. All of the equipment used was from the Affymetrix-Company (Affymetrix, High Wycombe, UK).

**Table 1:** Sequences of guide RNAs.

gRNAs	Sequence	Target
gRNA_TLR3_tm_up_fw	5’-aaacCCTCACTATCATGGGTTCCC-3’	TLR3 transm_up
gRNA_TLR3_tm_up_rev	5’-caccGGGAACCCATGATAGTGAGG-3’	TLR3 transm_up
gRNA_TLR3_tm_down_fw	5’-caccGTACTTCTCATCCACTTTGA-3’	TLR3 transm_down
gRNA_TLR3_tm_down_rev	5’-aaacTCAAAGTGGATGAGAAGTAC-3’	TLR3 transm_down
gRNA_TLR4_2a_fw	5’-caccGTCCAGGTTCTTGGTTGAGA-3’	TLR4 exon 2
gRNA_TLR4_2a_rv	5’-aaacTCTCAACCAAGAACCTGGAC-3’	TLR4 exon 2
gRNA_TLR4_2b_fw	5’-caccGATAAATCCAGCACCTGCAGTTCT-3’	TLR4 exon 2
gRNA_TLR4_2b_rev	5’-aaacAGAACTGCAGGTGCTGGATTTATC-3’	TLR4 exon 2
gRNA_TLR4_3a_fw	5’-caccGTCTAAAGAGAGATTGAGTA-3’	TLR4 exon 3
gRNA_TLR4_3a_rv	5’-aaacTACTCAATCTCTCTTTAGAC-3’	TLR4 exon 3
gRNA_TLR4_3b_fw	5’-caccGAAGTCCATCGTTTGGTTCT-3’	TLR4 exon 3
gRNA_TLR4_3b_rv	5’-aaacAGAACCAAACGATGGACTTC-3’	TLR4 exon 3

Lower case letters indicate overhangs compatible with BbsI cut sites.

**Table 2:** Sequences of primers.

Primer	Sequence
P3511_sgRNA_seq_fwd	5’-CTTGGGTAGTTTGCAGT-3’
P3511_sgRNA_seq_rev	5’-GAGCCATTTGTCTGCAG-3’
tlr4_exon2_fw	5’-CCATCTCTGGTCTAGGAGAGG-3’
tlr4_exon2_rev	5’-CAGCCAACTGCCTACTTCACAG-3’
tlr4_exon3_fw	5’-GACCAATCTAGAGCACTTGGAC-3’
tlr4_exon3_rev	5’-CAAGGCTTGGTAGATCAACTTCTG-3’
tlr3_tm_fw	5’-GCGCTTTAATCCCTTTGATTGC-3’
tlr3_tm_rev	5’-GCCTCAAAGTCCCTTTCTTCC-3’
tnf_fw	5’-GCTGCACTTTGGAGTGAT-3’
tnf_rev	5’-CCTCAGCTTGAGGGTTTG-3’
il-1β_fw	5’-CCAGTGAAATGATGGCTTATTAC-3’
il-1β_rev	5’-TAGTGGTGGTCGGAGATT-3’
IL6_qPCR_for	5’-TCAGCCCTGAGAAAGGA-3’
IL6_qPCR_rev	5’-TTTCACCAGGCAAGTCTC-3’
irak2_fw	5’-GGAAGCCATTCGTCTTCAA-3’
irak2_rev	5’-TCTTGCAGCACAGAAGC-3’
ifit2_fw	5’-GAGGAAGATTTCTGAAGAGTG-3’
ifit2_rev	5’-CAAGTTCCAGGTGAAATGGC-3’
ifi44_fw	5’-CCACCGTCAGTATTTGGAAT-3’
ifi44_rev	5’-GGGCCTATAGTCTCTGATGT-3’
gapdh_fw	5’-TTCACCACCATGGAGAAGG-3’
gapdh_rev	5’-TGGTGCAGGAGGCATTG-3’

### Bioinformatics and statistics

A Custom CDF Version 22 with ENTREZ based gene definitions was used to annotate the arrays^19^. The raw fluorescence intensity values were normalized applying quantile normalization and RMA background correction. ANOVA was performed to identify differentially expressed genes using a commercial software package SAS JMP10 Genomics, version 6, from SAS (SAS Institute, Cary, NC, USA). A false positive rate of a = 0.05 with FDR correction was taken as the level of significance. Adjusted *P*-values are listed in the tables showing induced genes.

**Table 3:** List of induced inflammatory genes 4 h post infection.

GeneSymbol	GeneName	Adjusted *P*-value for Diff of groups = (Chlamydia) - (Ctrl)
IRF9	interferon regulatory factor 9	8.726 × 10^− 06^
IFI44L	interferon induced protein 44 like	2.358 × 10^− 02^
IFI6	interferon alpha inducible protein 6	2.089 × 10^− 02^
ICAM1	intercellular adhesion molecule 1	3.366 × 10^− 07^
IFNA14	interferon alpha 14	3.714 × 10^− 02^
IL1A	interleukin 1 alpha	1.148 × 10^− 04^
IL1B	interleukin 1 beta	1.125 × 10^− 03^
IL6	interleukin 6	5.280 × 10^− 08^
IL11	interleukin 11	3.979 × 10^− 07^
IRAK2	interleukin 1 receptor associated kinase 2	1.193 × 10^− 08^
ITSN2	intersectin 2	2.334 × 10^− 02^
TNF	tumor necrosis factor	1.038 × 10^− 06^

All statistical analyses were performed using GraphPad Prism 8.3.0 (GraphPad Software, LLC). Statistical comparisons of two groups were analyzed by unpaired t test, of more than two groups by one- or two-way ANOVA, post hoc test Tukey, Sidak, or Dunnetts as indicated in individual figure legends. *P* values < 0.05 were considered as statistically significant.

### Generation of *tlr4-* or *tlr3*-deficient T24/83 cells

We used CRISPR/Cas9 to generate *tlr4-* or 3-deficient T24/83 cells. Briefly, we cloned two different gRNAs specific for exon 2 and two for exon 3 of TLR4 into the plasmid p3511 (Addgene plasmid #48138, kind gift from Dr. Georg Stoecklin, Division of Biochemistry, Med. Faculty Mannheim, Germany) using BbsI as restriction enzyme^20^. We used primers gRNA_TLR4_2a_fw, gRNA_TLR4_2a_rv, gRNA_TLR4_2b_fw, gRNA_TLR4_2b_rev, gRNA_TLR4_3a_fw, gRNA_TLR4_3a_rv, gRNA_TLR4_3b_fw, gRNA_TLR4_3b_rv to generate TLR4 exon 2- and TLR4 exon 3-specific gRNAs (Table 1). We verified the resulting four different plasmids (p3511+gRNA_TLR4_2a, p3511+gRNA_TLR4_2b, p3511+gRNA_TLR4_3a, p3511+gRNA_TLR4_3b) by restriction analysis with BbsI and EcoRV and PCR using the primers P3511_sgRNA_seq_fwd and P3511_sgRNA_seq_rev (Table 2) and sequencing for the correct integration of gRNAs. In case of TLR3, we used primers gRNA_TLR3_tm_up_fw, gRNA_TLR3_tm_up_rev, gRNA_TLR3_tm_down_fw, gRNA_TLR3_tm_down_rev (Table 1) to generate two different TLR3 transmembrane region-specific gRNAs and cloned them into the plasmid p3511+gRNA_TLR3_tm_up and p3511+gRNA_TLR3_tm_down. We verified both plasmids by restriction analysis with BbsI and EcoRV and PCR using the primers P3511_sgRNA_seq_fwd and P3511_sgRNA_seq_rev (Table 2) and sequencing for the correct integration of gRNAs.

In addition to the gRNAs, the plasmid p3511 encodes for Cas9 and GFP. We transfected the four plasmids in case of TLR4 and two plasmids in case of TLR3 into T24/83 cells using Lipofectamine 3000 and enriched GFP-expressing T24/83 cells two days after transfection via cell sorting. Single clones were obtained by limiting dilution cloning using 96-well plates and 10% (v/v) filtered conditioned medium from T24/83 cells. Growing cells were subsequently tested by PCR using the primers tlr4_exon2_fw, tlr4_exon2_rev, tlr4_exon3_fwd, tlr4_exon3_rev, tlr3_tm_fw, tlr3_tm_rev (Table 2) and sequencing of the PCR-products whether mutations were introduced into exon 2 and exon 3 of TLR4 and the transmembrane region of TLR3. The clones were further verified by cytokine analysis (Fig. S7, S8).

### Analysis of number and size of chlamydial inclusions

To determine chlamydial inclusion numbers, wild type, *tlr4*-, or *tlr3*-deficient T24/83 cells were seeded in 24-well plates on glass cover slips one day prior to further treatment. The cells were infected with *C. trachomatis* serovar D (MOI 1) for 2 h in medium without FCS. After two hours, the medium was changed to FCS-containing medium. 24 h p.i., we washed the infected cells three times with PBS to remove the medium. After fixation with a 1:1 mixture of acetone: methanol, we stained the cells with anti-Chlamydia Multiepitope Cocktail mouse monoclonal for 30 min at 37 °C. After removing the antibodies by washing the glass cover slips alternately with water and PBS, the samples were mounted in 1:1 glycerol/PBS and stored at 4 °C until imaging.

To examine the influence of TLR4 or TLR3 on the release of infectious progeny, we seeded wild type, *tlr4-*, or *tlr3-*deficient T24/83 cells in 24-well plates. The cells were infected with *C. trachomatis* serovar D (MOI 0.15) in medium without FCS. After two hours, we changed the medium, which now contained FCS. After further 46 h, we disrupted the T24/83 cells with glass beads, removed the cellular debris by centrifugation and incubated the *Chlamydia*-containing supernatants for 24 h with new wild type, *tlr4-*, or *tlr3-*deficient T24/83 cells. We washed the infected cells three times with PBS to remove the medium and performed the ACI-FITC staining as described above.

We determined the size of the chlamydial inclusions with Fiji.

### Quantification of the intracellular amount of the UPEC strain CFT073

We seeded 4*10^5^ T24/83 wild type, *tlr4-*, or *tlr3*-deficient cells in a 24-well plate. On the next day cells were infected with CFT073 with an MOI 1 or MOI 0.1. The plates were centrifuged for 5 min at 300 x g to bring bacteria in contact with the T24/83 cells. Afterwards the plates were incubated at 37 °C, 5% CO_2_ for 1 h. The medium was changed to gentamycin (400 µg/ml) containing infection medium (3% FCS). An incubation for 3 h followed. The supernatant was removed and cells were washed twice with PBS. 1 ml saponin (1%) was added for 10 min at RT, the lysed T24/83 cells were diluted 1:200 with PBS and 100 µl were plated on an agar plate. The plates were incubated at 37 °C. On the next day the colonies from intracellular *E. coli* were counted.

## Results

## Transcriptional pro-inflammatory response of the uroepithelial cell line T24/83 upon infection with *C. trachomatis* serovar D

**Fig. 1:**
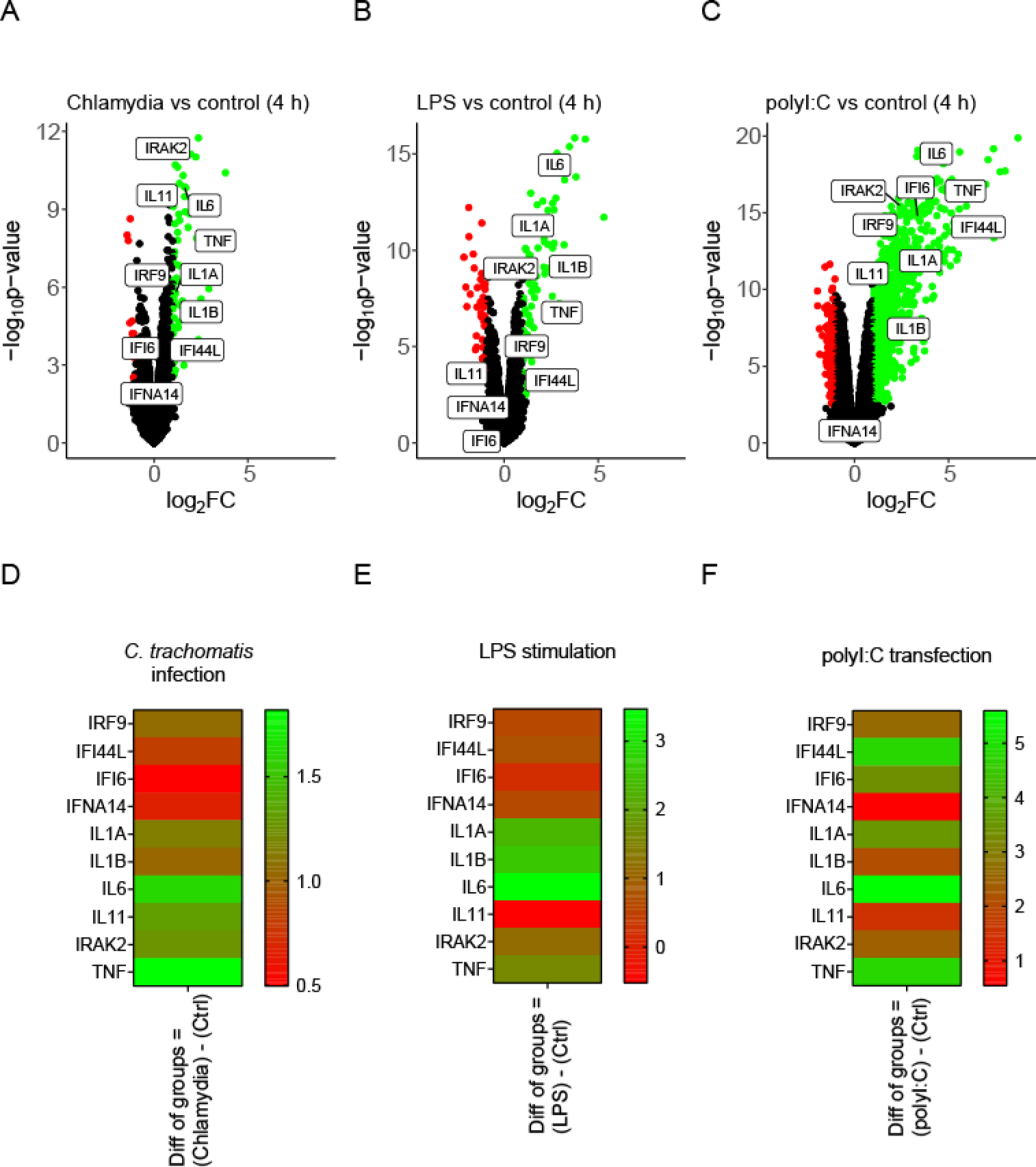
Early pro-inflammatory gene induction 4 h post infection with *C. trachomatis* serovar D. We infected T24/83 cells (5 × 10^5^ cells/well) with *C. trachomatis* serovar D (MOI 5, A, D) or stimulated them with LPS (1 µg/ml, B, E) or polyI:C/lipofectamine (5 µg/ml, C, F) for 4 h, analyzed their transcriptome by microarrays and depicted the results of infected or stimulated cells versus untreated cells as volcano plots (**A**-**C**). We selected interleukins, interferons, interferon-induced genes, tumor necrosis factor-α (TNF-α) and TNF-α-related genes which were significantly regulated (*P* < 0.05). In (**A**-**C**) green dots indicate genes with a fold induction of ≥ 1, red dots genes with a fold induction of ≤ -1, and black dots all genes with a fold induction between these values. (**D**-**F**) depict heat maps of the selected and significantly regulated pro-inflammatory genes, depicting either *C. trachomatis* serovar D infected cells (**D**), LPS-stimulated cells (**E**) or polyI:C/lipofectamine transfected cells (**F**). Table 3 lists identified gene names.

**Fig. 2:**
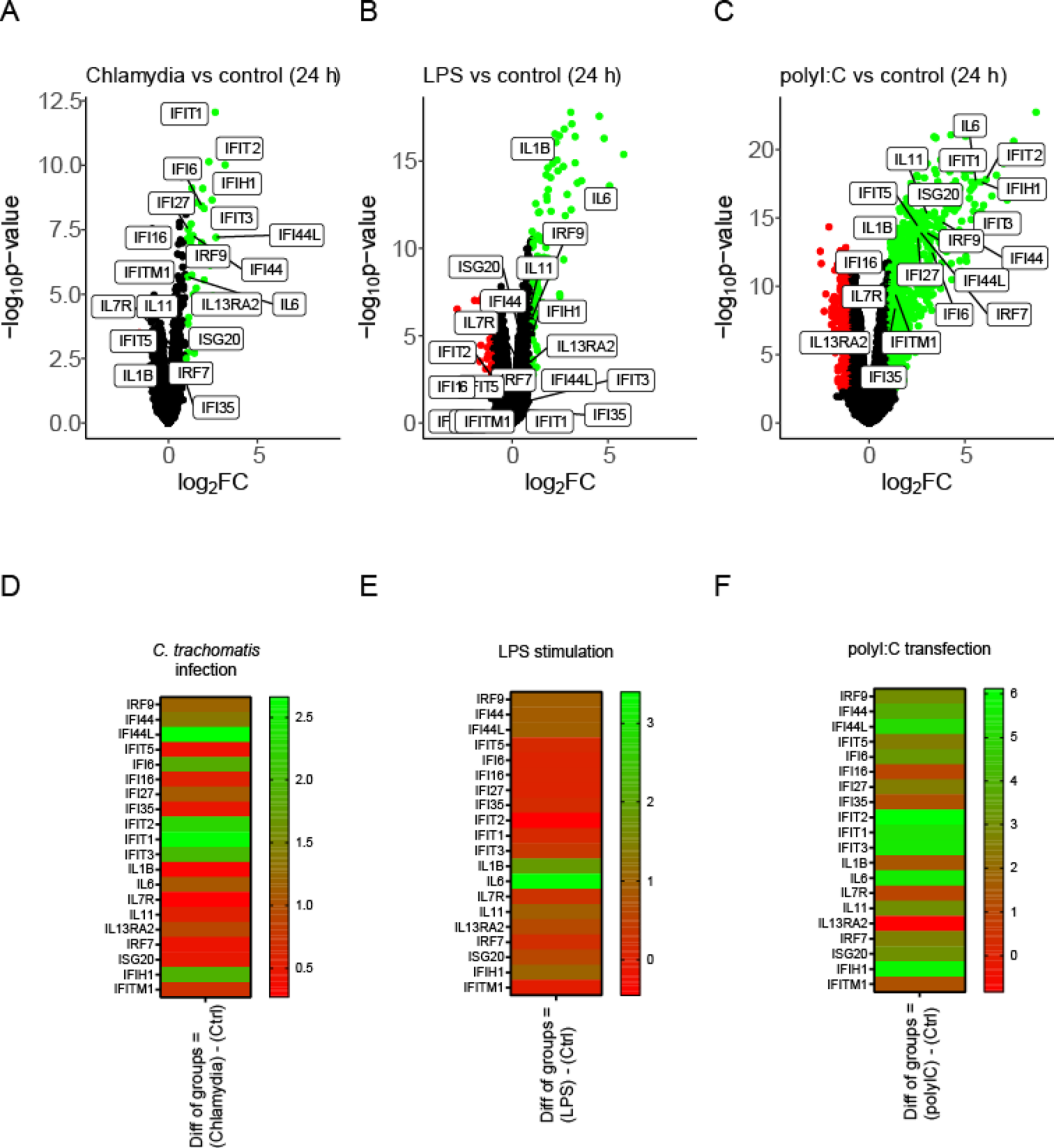
Late pro-inflammatory gene induction 24 h post infection with *C. trachomatis* serovar D. We infected T24/83 cells (5 × 10^5^ cells/well) with *C. trachomatis* serovar D (MOI 5, A, D) or stimulated them with LPS (1 µg/ml, B, E) or polyI:C/lipofectamine (5 µg/ml, C, F) for 24 h, analyzed their transcriptome by microarrays and depicted the results of infected or stimulated cells versus untreated cells as volcano plots (**A**-**C**). We selected interleukins, interferons, interferon-induced genes, tumor necrosis factor-α (TNF-α) and TNF-α-related genes which were significantly regulated (*P* < 0.05). In (**A**-**C**) green dots indicate genes with a fold induction of ≥ 1, red dots genes with a fold induction of ≤ -1, and black dots all genes with a fold induction between these values. (**D**-**F**) depict heat maps of the selected and significantly regulated pro-inflammatory genes, depicting either *C. trachomatis* serovar D infected cells (**D**), LPS-stimulated cells (**E**) or polyI:C/lipofectamine transfected cells (**F**). Table 4 lists identified gene names.

As pointed out above we selected the human uroepithelial cancer cell line T24/83 for the analysis of its interaction with *C. trachomatis* serovar D not only because it allowed the complete replication of the pathogen and expressed several PRRs but also due to its best IFN-β response upon polyI: C-transfection (Fig. S1). We considered this presumably cytosolic helicase-driven response as an important marker for the initiation of inflammation by this obligate intracellular pathogen^11^. We then monitored the transcriptional pro-inflammatory response of this cell line post infection with *C. trachomatis* serovar D using microarrays. We focused on genes encoding for interleukins, interferons, interferon-induced genes, tumor necrosis factor-α (TNF-α) and TNF-α-related genes, and explored whether their expression changes significantly 4–24 h post infection. We found significantly induced pro-inflammatory genes such as TNF-α, IL-6 and others 4 h post infection (Fig. 1A, D; Table 3). The pattern of induced genes differed from the ones provoked by LPS-stimulation (Fig. 1B, E) or polyI:C/lipofectamine transfection (Fig. 1C, F). The picture of induced pro-inflammatory genes changed 24 h post infection with *C. trachomatis* serovar D as interferon-related genes dominated (Fig. 2A, D; Table 4). LPS-stimulation induced this set of genes rather weakly (Fig. 2B, E) while polyI:C/lipofectamine transfection elicited a profound response, as expected (Fig. 2C, F). Taken together, the expression of cytokine genes induced by *C. trachomatis* serovar D was comparatively weak and the expression pattern differed considerably from the pattern induced by LPS-stimulation or polyI:C/lipofectamine transfection, although genes induced by all three conditions such as IL-6 and TNF-α could be identified (Fig. 1).

**Table 4:** List of induced inflammatory genes 24 h post infection.

GeneSymbol	GeneName	Adjusted *P*-value for Diff of groups = (Chlamydia) - (Ctrl)
IRF9	interferon regulatory factor 9	3.597 × 10^− 06^
IFI44	interferon induced protein 44	3.597 × 10^− 06^
IFI44L	interferon induced protein 44 like	3.997 × 10^− 06^
IFIT5	interferon induced protein with tetratricopeptide repeats 5	2.001 × 10^− 02^
IFI6	interferon alpha inducible protein 6	4,034 × 10^− 07^
ICAM1	intercellular adhesion molecule 1	1.485 × 10^− 04^
IFI16	interferon gamma inducible protein 16	6.567 × 10^− 06^
IFI27	interferon alpha inducible protein 27	2,211 × 10^− 06^
IFI35	interferon induced protein 35	2.439 × 10^− 02^
IFIT2	interferon induced protein with tetratricopeptide repeats 2	1.577 × 10^− 08^
IFIT1	interferon induced protein with tetratricopeptide repeats 1	3.848 × 10^− 10^
IFIT3	interferon induced protein with tetratricopeptide repeats 3	4.877 × 10^− 07^
IL1B	interleukin 1 beta	2.055 × 10^− 02^
IL6	interleukin 6	7.500 × 10^− 05^
IL7R	interleukin 7 receptor	1.469 × 10^− 02^
IL11	interleukin 11	2.965 × 10^− 03^
IL13RA2	interleukin 13 receptor subunit alpha 2	3.606 × 10^− 04^
IRF7	interferon regulatory factor 7	2.329 × 10^− 02^
ISG20	interferon stimulated exonuclease gene 20	1.066 × 10^− 02^
IFIH1	interferon induced with helicase C domain 1	1.108 × 10^− 07^
IFITM1	interferon induced transmembrane protein 1	1.447 × 10^− 04^

**Table 5:** List of *C. trachomatis* serovar D induced genes 4 h post infection.

GeneSymbol	GeneName	Adjusted *P*-value for Diff of groups = (Chlamydia) - (Ctrl)
MMP1	matrix metalloproteinase 1	1.813 × 10^− 08^
MMP3	matrix metalloproteinase 3	8.247 × 10^− 05^
HTN1	histatin 1	4.613 × 10^− 04^
KRTAP4-12	keratin associated protein 4–12	1.707 × 10^− 04^
HTN3	histatin 3	3.063 × 10^− 03^
PTGS2	prostaglandin-endoperoxide synthase 2	5.355 × 10^− 09^
CXCL3	C-X-C motif chemokine ligand 3	3.900 × 10^− 07^
TNF	tumor necrosis factor	1.038 × 10^− 06^
STATH	statherin	4.106 × 10^− 03^
TNFAIP3	TNF alpha induced protein 3	5.708 × 10^− 08^
ICAM1	intercellular adhesion molecule 1	3.366 × 10^− 07^
SEMA7A	semaphorin 7 A (John Milton Hagen blood group)	3.033 × 10^− 09^
RGS2	regulator of G protein signaling 2	2.233 × 10^− 08^
CXCL2	C-X-C motif chemokine ligand 2	1.974 × 10^− 04^
SLC2A3	solute carrier family 2 member 3	2.788 × 10^− 04^
CD69	CD69 molecule	1.039 × 10^− 02^
LIPG	lipase G, endothelial type	2.630 × 10^− 04^
CSF3	colony stimulating factor 3	6.282 × 10^− 07^
SERPINE1	serpin family E member 1	4.064 × 10^− 08^
INSIG1	insulin induced gene 1	1.652 × 10^− 05^
HIC1	HIC ZBTB transcriptional repressor 1	8.544 × 10^− 04^
MMP10	matrix metalloproteinase 10	4.915 × 10^− 05^
ADM	adrenomedullin	2.720 × 10^− 06^

**Table 6:** List of *C. trachomatis* serovar D induced genes 24 h post infection.

GeneSymbol	GeneName	Adjusted *P*-value for Diff of groups = (Chlamydia) - (Ctrl)
HTN1	histatin 1	2,013 × 10^− 08^
IFIT1	interferon induced protein with tetratricopeptide repeats 1	3.848 × 10^− 10^
HTN3	histatin 3	3.145 × 10^− 04^
STATH	statherin	2.611 × 10^− 07^
MX1	MX dynamin like GTPase 1	3.111 × 10^− 05^
IFIT3	interferon induced protein with tetratricopeptide repeats 3	4.877 × 10^− 07^
IFIH1	interferon induced with helicase C domain 1	1.108 × 10^− 07^
IFI6	interferon alpha inducible protein 6	4.034 × 10^− 07^
OASL	2_-5_-oligoadenylate synthetase like	5.924 × 10^− 06^
RSAD2	radical S-adenosyl methionine domain containing 2	2.822 × 10^− 05^
CEACAM1	carcinoembryonic antigen related cell adhesion molecule 1	1.754 × 10^− 04^
HERC5	HECT and RLD domain containing E3 ubiquitin protein ligase 5	3.140 × 10^− 07^
OAS2	2_-5_-oligoadenylate synthetase 2	2.927 × 10^− 05^
C9orf106	chromosome 9 open reading frame 106 (putative)	2.264 × 10^− 02^
XAF1	XIAP associated factor 1	2.852 × 10^− 04^
IFI44	interferon induced protein 44	3.597 × 10^− 06^
CCL5	C-C motif chemokine ligand 5	1.515 × 10^− 05^
OAS1	2_-5_-oligoadenylate synthetase 1	6.426 × 10^− 05^
HERC6	HECT and RLD domain containing E3 ubiquitin protein ligase family member 6	1.077 × 10^− 07^

### *C. trachomatis* serovar D infection does not activate pro-inflammatory signaling cascades

**Fig. 3:**
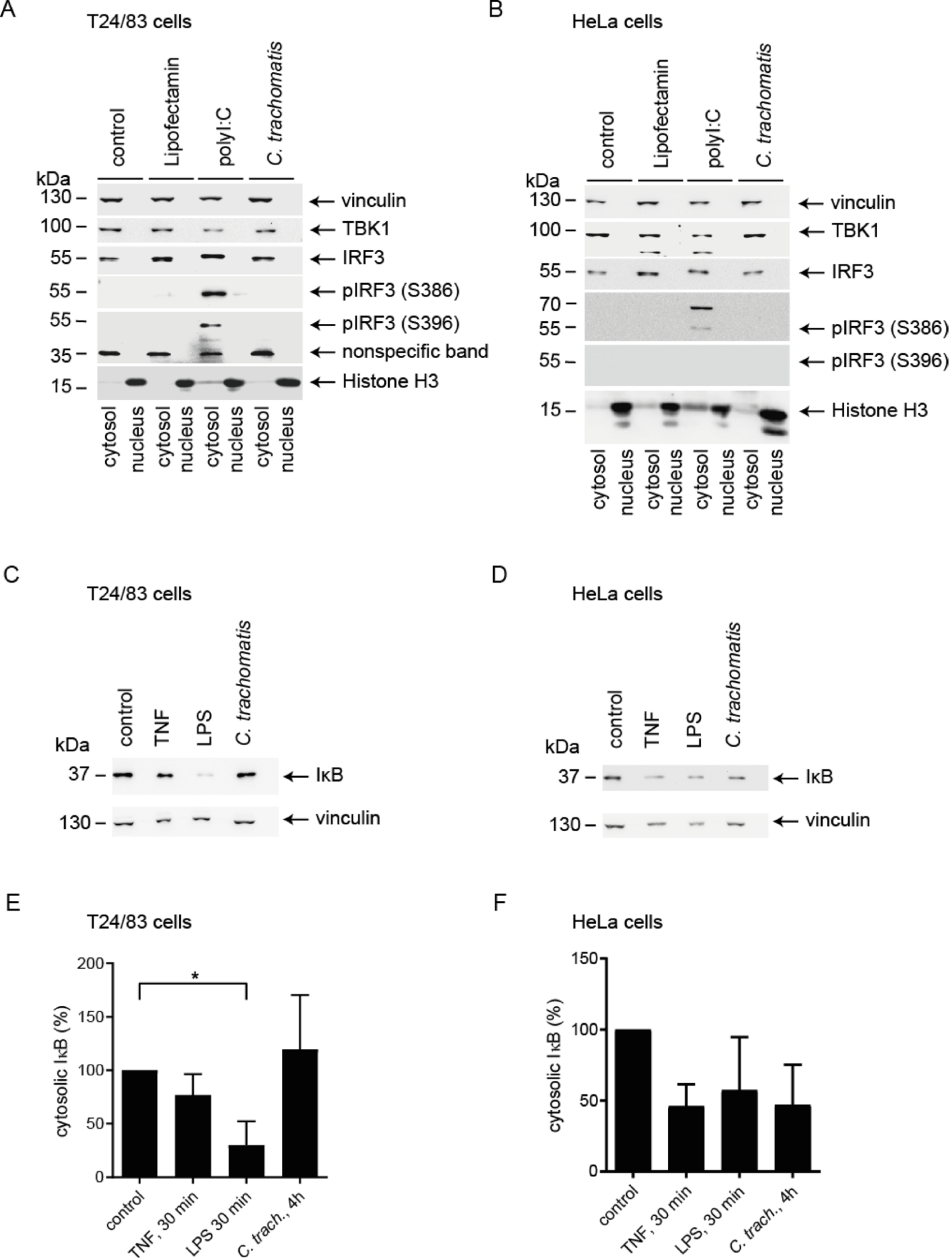
*C. trachomatis* serovar D fails to induce phosphorylation of IFR3 or degradation of IκB. In (**A**) and (**B**) untreated, lipofectamine treated (4 h), lipofectamine plus polyI:C (5 µg/ml, 4 h) stimulated or *C. trachomatis* serovar D infected (MOI 2, 4 h) T24/83 (5 × 10^5^ cells/well, A) or HeLa cells (5 × 10^5^ cells/well, B) were lysed with Nonidet buffer and the remaining pellet with Laemmli buffer to prepare cytosolic and nuclear lysates, respectively. We separated the lysates by SDS-PAGE. We used antibodies specific for TBK1, IRF3, pIRF3 S386, pIRF3 S396, the latter two to detect phosphorylation of IRF3. In addition to the depicted experiments, the experiments were repeated once with similar results. In (C-F) the cytosolic lysate of untreated, TNF-α (1 ng/ml, 30 min.) or LPS (1 µg/ml, 30 min.) stimulated or *C. trachomatis* serovar D infected (MOI 2, 4 h) T24/83 (**C**, **E**) or HeLa cells (**D**, **F**) was prepared. We used antibodies specific for IκB to analyze degradation of IκB. Detection of vinculin or histone H3 served as loading control for the cytosolic or nuclear lysates, respectively. (**E**) and (**F**) represent the data of three independent experiments depicted in (**C**) and (**D**). We calculated the ratio of the density values of the IκB and vinculin band of each lane. We then normalized individual ratios to the unstimulated control, which was set to 100% in order to compare the three different experiments. The bars in (**E**) and (**F**) depict the calculated mean percentages +/- SD. *one way ANOVA, post hoc Dunnetts, *P* < 0.05. Individual blots were cropped from different parts of the same blot or from different blots.

We next examined whether *C. trachomatis* serovar D inclusions trigger key downstream components of pro-inflammatory-signaling cascades such as phosphorylation of IFN regulatory factor (IRF) 3 and degradation of IκB. We found that the infection of T24/83 and, in addition, HeLa cells with *C. trachomatis* serovar D induced neither the phosphorylation of IRF3 (Fig. 3A, B) nor the degradation of IκB (Fig. 3C-F). However, transfection of T24/83 cells with polyI:C/lipofectamine induced the phosphorylation of IRF3 at S386 and S396 while polyI:C/lipofectamine transfection of HeLa cells phosphorylated IRF3 only at S386 (Fig. 3A, B). At the time point analyzed, we did not observe that IRF3 translocated to the nucleus (Fig. 3A, B). Stimulation of T24/83 cells with endotoxin but not with TNF-α resulted in a significant IκB-degradation (Fig. 3C, E). HeLa cells did not respond significantly to both stimuli (Fig. 3D, F). These findings indicate that T24/83 and HeLa cells sensed *C. trachomatis* serovar D in a way not leading to a detectable activation of these signaling cascades.

**Fig. 4:**
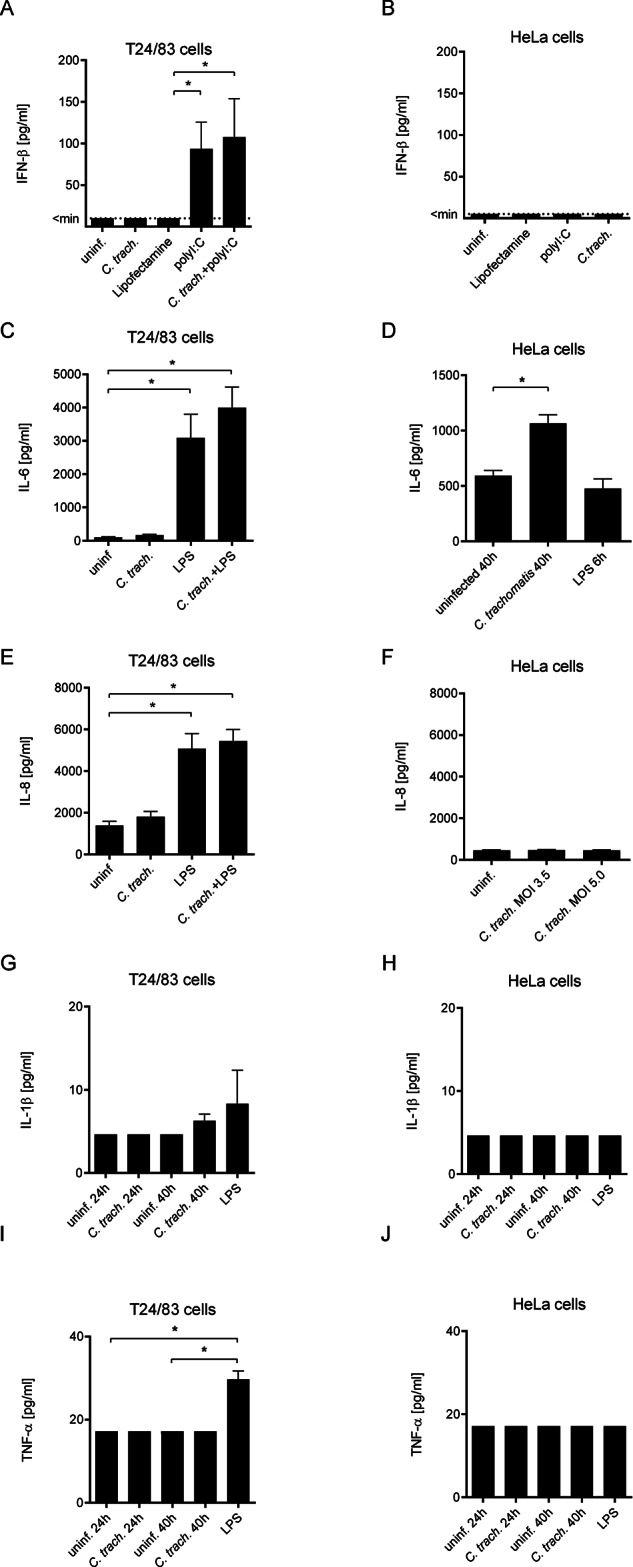
*C. trachomatis* serovar D neither induces pro-inflammatory cytokines (with the exception of IL-6 in HeLa cells) nor interferes with polyI:C/lipofectamine or LPS-induced cytokine secretion. We infected T24/83 (5 × 10^5^ cells/well) or HeLa cells (5 × 10^5^ cells/well) with *C. trachomatis* serovar D (MOI 5 or as indicated) for 24 h or as indicated in the individual graphs. We also stimulated the cells with LPS (1 µg/ml) or polyI:C (5 µg/ml)/lipofectamine or combined *C. trachomatis* serovar D infection with LPS or polyI:C/lipofectamine stimulation as indicated in the graphs. We measured IFN-β (**A**, **B**), IL-6 (**C**, **D**), IL-8 (**E**, **F**), IL-1β (**G**, **H**) and TNF-α (**I**, **J**) in the culture supernatant. In addition to the depicted experiments, the experiments were repeated twice with similar results. *one way ANOVA, post hoc Tukey, *P* < 0.05.

**Fig. 5:**
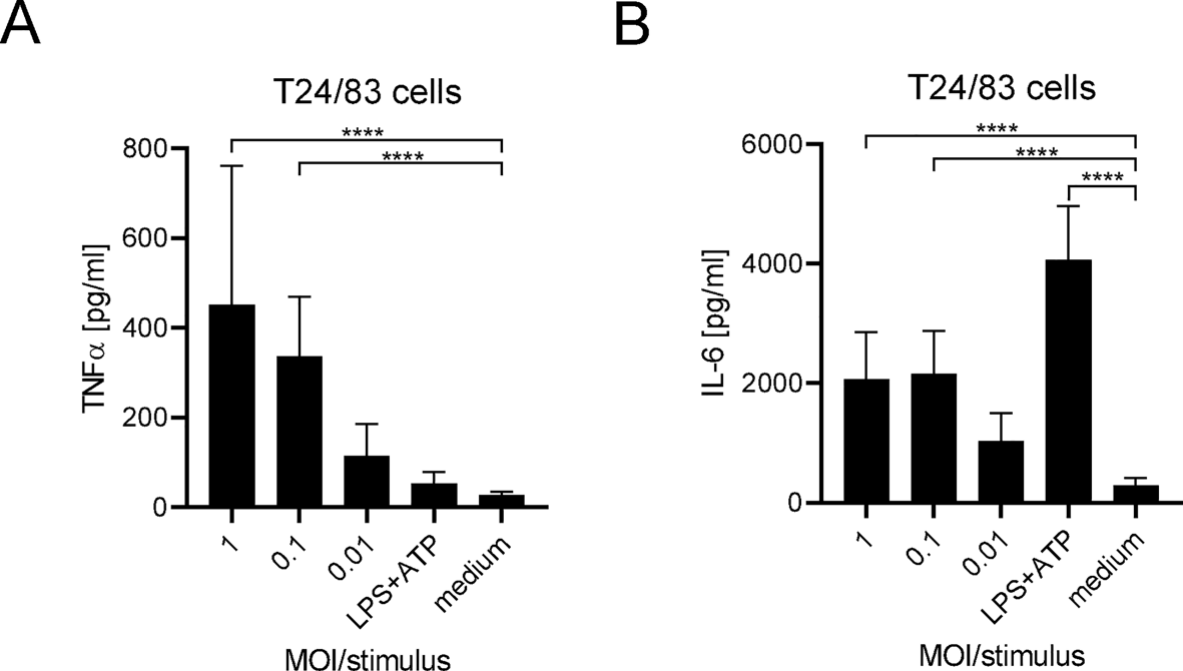
Infection with the *E. coli* strain CFT073 induces the secretion of pro-inflammatory cytokines by T24/83 cells. We infected T24/83 (4 × 10^5^ cells/well) with titrated amounts of the uropathogenic *E. coli* CFT073 as indicated in the graphs. IL-6 (**A**) and TNF-α levels (**B**) were determined 5 h post infection. We also stimulated the cells with LPS (100 ng/ml) plus ATP (5mM) as positive control. Graphs demonstrate four independent experiments. **** one way ANOVA, post hoc Tukey, *P* < 0.0001.

To verify these findings, we analyzed cytokine release by T24/83 and HeLa cells post infection with *C. trachomatis* serovar D. Infected T24/83 cells did not produce significantly different levels of IFN-β (Fig. 4A), IL-6 (Fig. 4C), IL-8 (Fig. 4E), IL-1β (Fig. 4G) and TNF-α (Fig. 4I) compared to non-infected cells. We obtained the same result with infected HeLa cells (Fig. 4B, F, H, J) with the exception of IL-6, which was significantly induced by the infection with *C. trachomatis* serovar D (Fig. 4D). While T24/83 cells responded to lipofectamine-transfected polyI:C, presumably triggering cytosolic helicases, with significantly increased IFN-β release (Fig. 4A) as well as to endotoxin with significantly elevated IL-6 (Fig. 4C), IL-8 (Fig. 4E) and TNF-α levels (Fig. 4I), HeLa cells reacted neither to lipofectamine-transfected polyI:C nor to endotoxin (Fig. 4B, D, F, H, J). Interestingly in the case of T24/83 cells, *C. trachomatis* serovar D infection did not prevent polyI:C/lipofectamine or endotoxin induced cytokine secretion (Fig. 4A, C, E). However, the pathogen impaired significantly polyI:C/dotap induced secretion of IFN-β (Fig. S2). Dotap transfection was described to be less efficient than lipofectamine transfection, since it does not avoid the active intracellular transport along microtubules and subsequent entrapment and degradation of the transfected material within lysosomal compartments^21^. But within the lysosomal compartment polyI:C/dotap may be sensed by another pattern recognition receptor such as TLR3 in contrast to polyI:C/lipofectamine. We also explored whether T24/83 cells recognize another pathogen interacting with uroepithelial cells, i.e. the uropathogenic *Escherichia coli* strain CFT073. The infection with titrated amounts of CFT073, including an MOI as low as 0.01, resulted in readily detectable secretion of IL-6 and TNF-α after five hours post infection (Fig. 5). Thus, T24/83 cells are able to secrete key pro-inflammatory cytokines upon activation with classical pathogen-associated molecular patterns (PAMPs) or the *E. coli* strain CFT073 but not upon infection with *C. trachomatis* serovar D.

### *C. trachomatis* serovar D induced transcriptome differs from endotoxin- or polyI: C-induced gene expression patterns

**Fig. 6:**
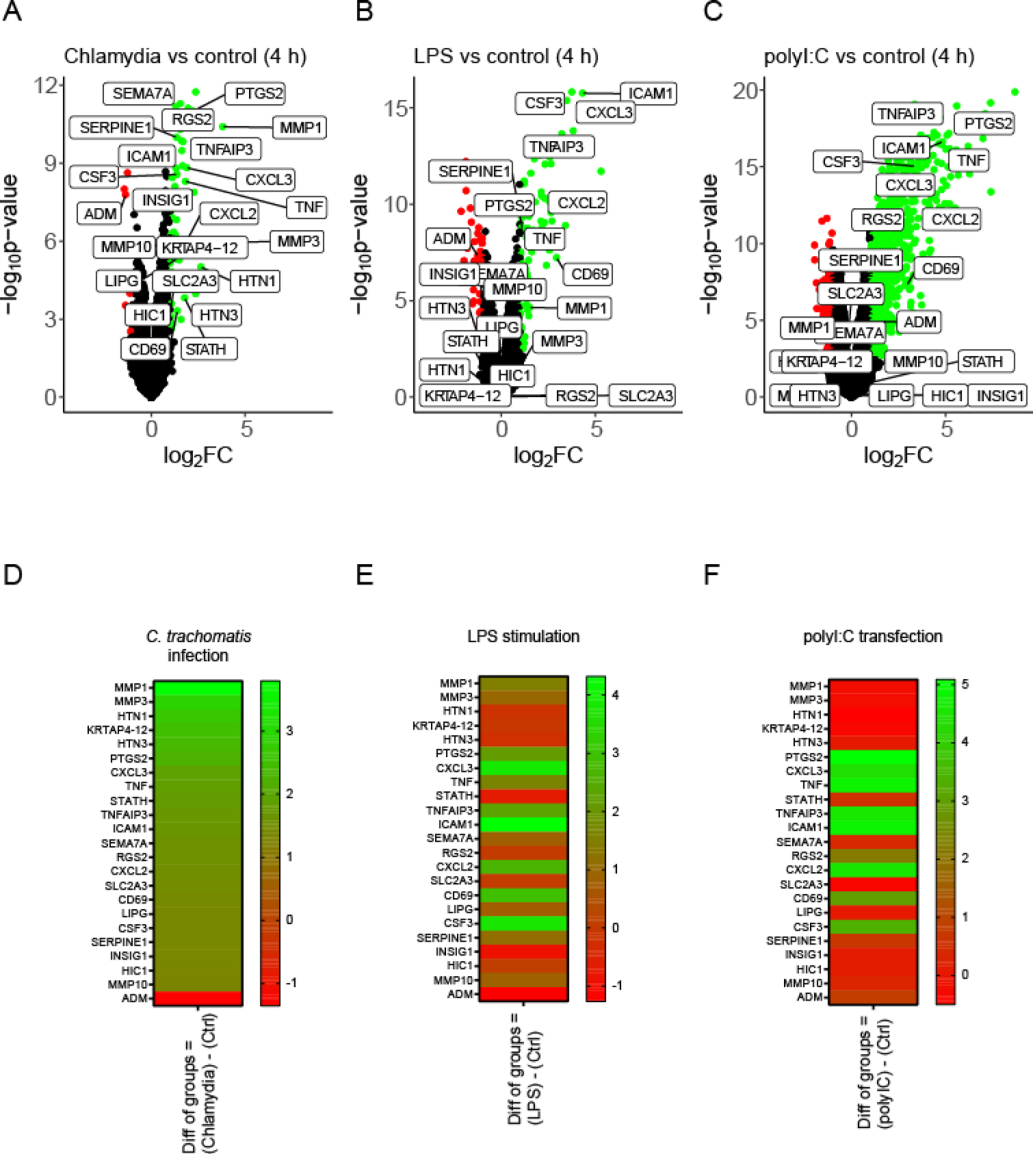
Early *C. trachomatis* serovar D induced genes. We evaluated the microarray experiment described in Fig. 1 for genes, which were significantly regulated by the infection with *C. trachomatis* serovar D (*P* < 0.05). We infected T24/83 (5 × 10^5^ cells/well) with *C. trachomatis* serovar D (MOI 5, A, D) or stimulated them with LPS (1 µg/ml, B, E) or polyI:C/lipofectamine (5 µg/ml, C, F) for 4 h, analyzed their transcriptome by microarrays and depicted the results of infected or stimulated cells versus untreated cells as volcano plots (**A**-**C**). In (**A**-**C**) green dots indicate genes with a fold induction of ≥ 1, red dots genes with a fold induction of ≤ -1, and black dots all genes with a fold induction between these values. The heat map in (**D**) depicts the selected and significantly regulated genes in *C. trachomatis* serovar D infected cells. Heat maps in (**E**) and (**F**) display the same set of genes depicted in (**D**) in LPS-stimulated cells (**E**) or polyI:C/lipofectamine stimulated cells (**F**). Table 5 lists identified gene names.

**Fig. 7:**
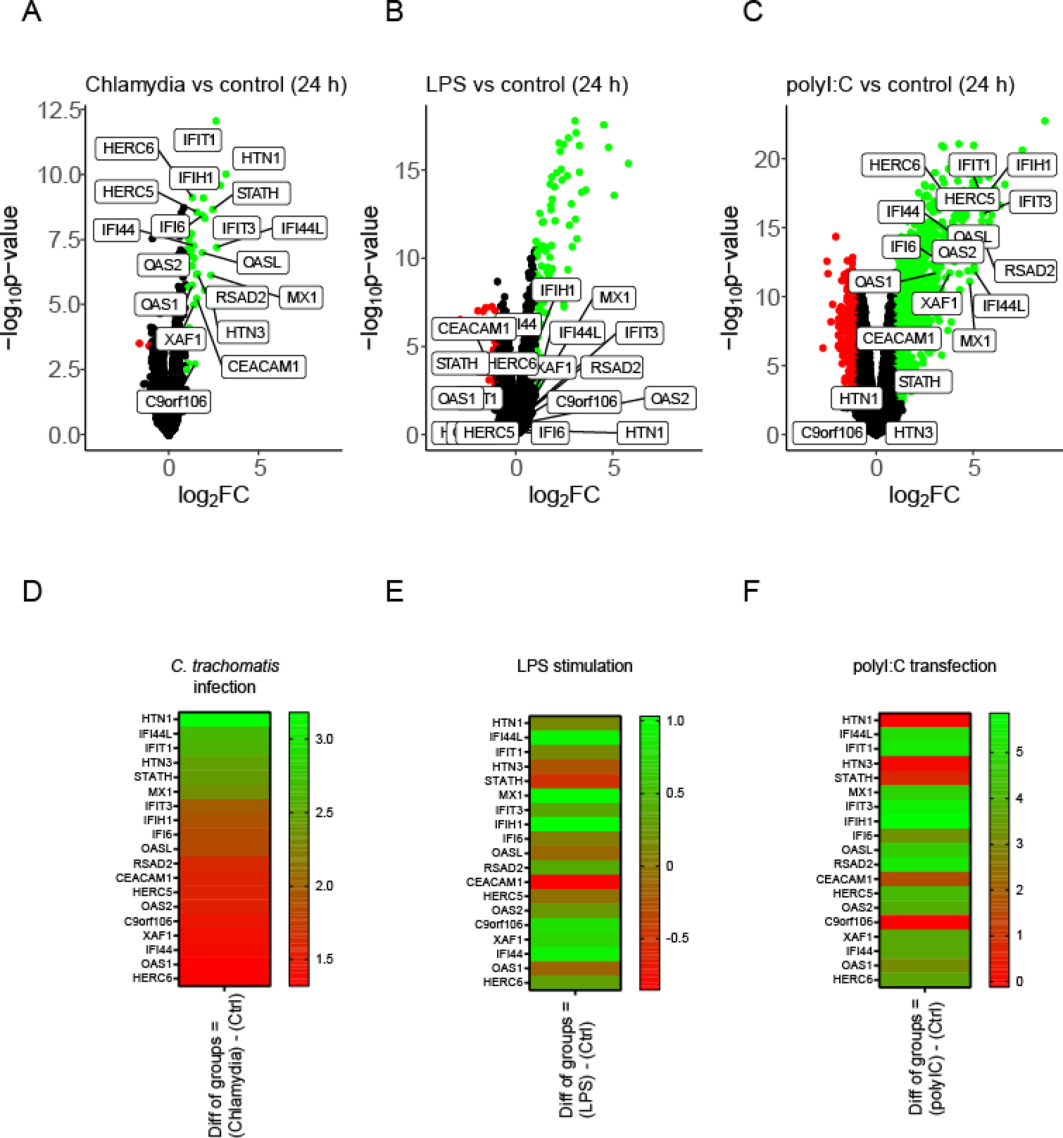
Late *C. trachomatis* serovar D induced genes. We evaluated the microarray experiment described in Fig. 2 for genes, which were significantly regulated by the infection with *C. trachomatis* serovar D (*P* < 0.05). We infected T24/83 (5 × 10^5^ cells/well) with *C. trachomatis* serovar D (MOI 5, A, D) or stimulated them with LPS (1 µg/ml, B, E) or polyI:C/lipofectamine (5 µg/ml, C, F) for 24 h, analyzed their transcriptome by microarrays and depicted the results of infected or stimulated cells versus untreated cells as volcano plots (**A**-**C**). In (**A**-**C**) green dots indicate genes with a fold induction of ≥ 1, red dots genes with a fold induction of ≤ -1, and black dots all genes with a fold induction between these values. The heat map in (**D**) depicts the selected and significantly regulated genes in *C. trachomatis* serovar D infected cells. Heat maps in (**E**) and (**F**) display the same set of genes depicted in (**D**) in LPS-stimulated cells (**E**) or polyI: C/lipofectamine stimulated cells (**F**). Table 6 lists identified gene names.

To investigate how T24/83 cells respond to the infection with *C. trachomatis* serovar D, we explored the most strongly and significantly induced genes four hours post infection with the pathogen. We found that the most induced genes were *mmp1* (matrix metallopeptidase 1), *mmp3* (matrix metallopeptidase 3), *htn1* (histatin 1), *krtap4-12* (keratin associated protein 4–12), *htn3* (histatin 3) and others (Fig. 6A, D). Some genes of this subset were induced by LPS (Fig. 6B, E) and polyI:C/lipofectamine (Fig. 6C, F). Interestingly, the most prominent genes induced by *C. trachomatis* serovar D were not or much weaker induced by both PAMPs (Fig. 6D-F). Twenty-four hours p.i., the gene subset induced by *C. trachomatis* serovar D changed considerably. While *htn1* and *htn3* were still prominent, interferon induced genes and others emerged (Fig. 7A, D). This subset of genes was only weakly induced by LPS (Fig. 7B, E), but strongly by polyI:C (Fig. 7C, F). Both PAMPs induced the expression of *htn1* not or only weakly, while *C. trachomatis* serovar D infected cells expressed this gene the most (Fig. 7D-F). These data indicate that the consequence of *C. trachomatis* serovar D recognition differs considerably from LPS or polyI:C/lipofectamine.

### Development of *C. trachomatis* serovar D inclusions in *tlr4-* or *tlr3-*deficient T24/83 cells

**Fig. 8:**
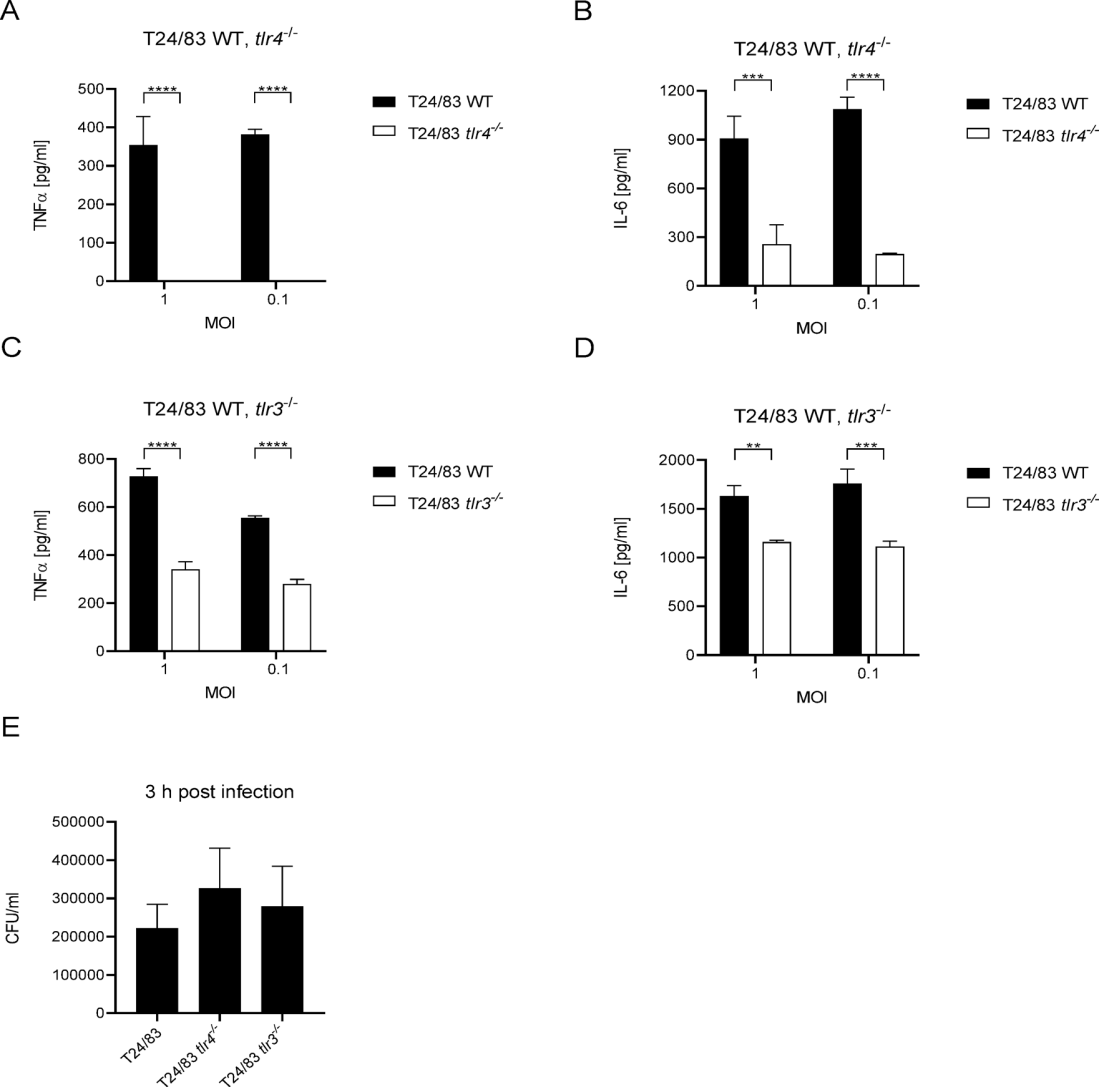
T24/83 cells sense the *E. coli* strain CFT073 in a TLR4- and TLR3-dependent manner. We infected wild type, *tlr4-* or *tlr3-*deficient T24/83 cells with CFT073 with MOIs indicated and determined their ability to secrete TNF-α (**A**, **C**) and IL-6 (**B**, **D**). Further, we evaluated the number of intracellular CFT073 in wild type and TLR-deficient T24/83 cell lines (**E**). **, ***, **** one way ANOVA, post hoc Tukey, *P* < 0.01, 0.001, < 0.0001, respectively.

T24/83 cells express a number of pattern-recognition receptors such as TLR4, TLR3 or cytosolic helicases as indicated by their pro-inflammatory response to LPS and polyI:C. Indeed, we detected the expression of *tlr3* and *ticam1* by reverse transcription PCR in T24/83 as well as HeLa cells (Fig. S3). We did not detect TLR2 by Western blot (Fig. S4). Since TLR3 was described to be involved in the recognition of *C. trachomatis* in epithelial cells^11^ and TLR4 to be expressed in fallopian tube epithelial cells^22^, we wondered whether these TLRs influence the replication of *C. trachomatis* serovar D in T24/83 cells. Moreover, we and others published earlier that purified and synthetic endotoxin of *C. trachomatis* is a weak TLR4 agonist^23–25^. Finally, genes of the Toll-like receptor signaling pathway were enriched in T24/83 cells 4 h (including TLR4) and 24 h post infection (including TLR3) with *C. trachomatis* serovar D (Fig. S5, S6). We, therefore, generated *tlr4-*deficient T24/83 cells using CRISPR/Cas9. We targeted exon 2 and exon 4 of TLR4 and *tlr4-*deficient cells displayed a deletion in exon 2 in both alleles and a deletion in exon 4 also in both alleles (Fig. S7A). *Tlr4-*deficient T24/83 cells did not secrete IL-6 upon stimulation with LPS (Fig. S7B). We also targeted the transmembrane region in exon 4 of TLR3 and obtained a deletion of both alleles in this region (Fig. S8A). Dotap-transfected polyI:C (delivering polyI:C to endosomes) stimulated T24/83 cells, if they were stimulated with IFN-γ which presumably increased TLR3 expression (Fig. S8B). IFN-α was less efficient. The response of IFN-γ stimulated *tlr3-*deficient cells to dotap-transfected polyI:C was clearly and significantly reduced (Fig. S8B). We also evaluated the response of *tlr4-* or *tlr3-*deficient T24/83 cells to an infection with CFT073. As shown in Fig. 8A and B, TNF-α and IL-6 secretion of infected *tlr4-*deficient cells was largely attenuated, although the number of intracellular CFT073 was slightly higher in *tlr4-*deficient T24/83 cells (Fig. 8E). Similarly, *tlr3-*deficient T24/83 cells displayed an impaired cytokine secretion upon CFT073 infection, while the intracellular CFT073 burden was again slightly increased (Fig. 8C-E). These findings indicate that the TLR-deficient T24/83 cell lines behaved as expected.

**Fig. 9:**
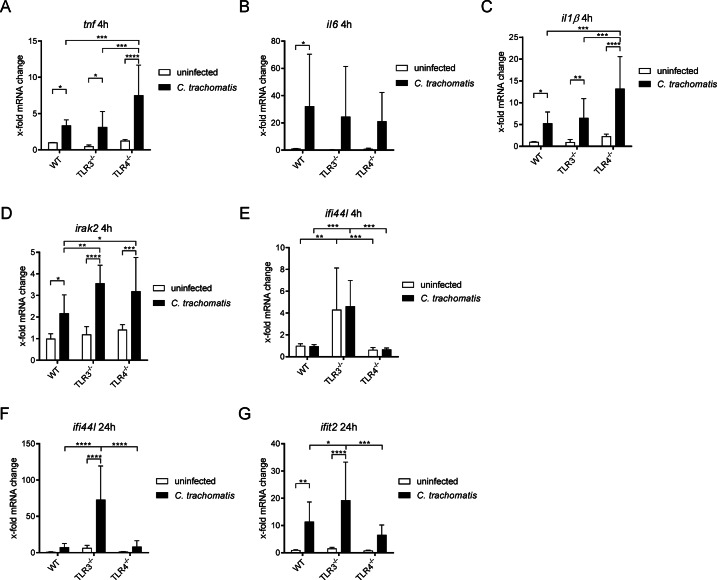
Influence of TLR3 and TLR4 on the transcription of pro-inflammatory genes post infection with *C. trachomatis* serovar D. We infected wild type or *tlr3-* or *tlr4-*deficient T24/83 cells (5 × 10^5^ cells/well) with *C. trachomatis* serovar D (MOI 5) for 4–24 h as indicated in the graphs. We used uninfected cells as controls. We isolated the cellular RNA and performed a reverse transcription real time PCR. We used the primers listed in Table 2 to detect *tnf* (**A**), *il6* (**B**), *il1β* (**C**), *irak2* (**D**), *ifi44l* (**E**, **F**) and *ifit2* (**G**) mRNAs. We calculated the relative fold gene expression of samples as described in methods. Bars in each graph depicts the mean ± standard deviation of three independent experiments, each experiment was performed with three technical replicates. *, **, ***, **** two way ANOVA, post hoc Sidak, *P* < 0.05, < 0.01, < 0.001, < 0.0001, respectively.

We used these cells to evaluate whether TLR3 or TLR4 are involved in the induction of transcription of pro-inflammatory genes post *C. trachomatis* serovar D infection identified in the microarray analysis (Figs. 1 and 2). We confirmed by reverse transcription real time PCR that the infection with *C. trachomatis* serovar D (MOI 5) induced the transcription of *tnf*, *il6*, *il1β*, *irak2* and *ifit2* significantly (Fig. 9A-D, G). In contrast, *ifi44l* was not induced (Fig. 9E, F). We did observe significant differences of *tlr4-* and *tlr3-*deficient T24/83 cells compared to wild type cells post infection. Thus, *tlr4-*deficient T24/83 cells demonstrated a significant higher *tnf*,* il1β* and *irak2* expression (Fig. 9A, C, D), while *tlr3-*deficient T24/83 cells showed a significant higher expression of *irak2*, *ifi44l* (24 h) and *ifit2* post *C. trachomatis* serovar D infection (Fig. 9D, F, G). It thus appears that TLR3 and TLR4 suppressed certain cytokines. The interpretation of these findings is presently unclear and requires further experiments. Infection of wild type, *tlr3-* and *tlr4-*deficient T24/83 cells with a lower dose of *C. trachomatis* serovar D (MOI 3) resulted partially in similar trends of cytokine expression. However, we found no significant differences between *tlr4-*, *tlr3-*deficient and wild type T24/83 cells in cytokine expression induced by *C. trachomatis* serovar D (Fig. S9).

**Fig. 10:**
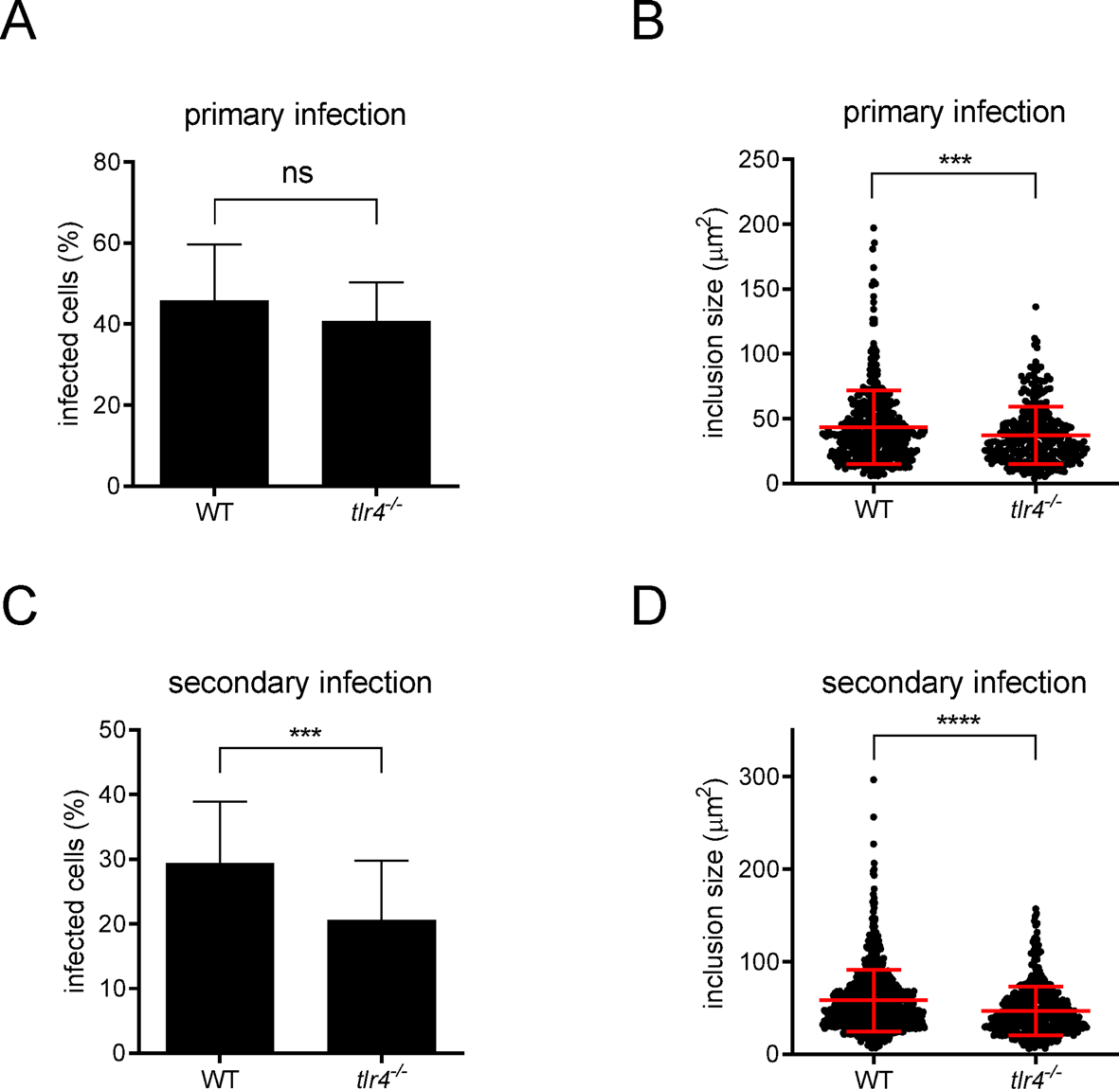
TLR4-dependent development of *C. trachomatis* serovar D inclusions. We infected wild type or *tlr4-*deficient T24/83 cells (2.5 × 10^5^ cells/well) with *C. trachomatis* serovar D (MOI 1) for 24 h. We fixed, permeabilized and stained the cells with an ACI-FITC anti-chlamydia antibody to detect chlamydial inclusions. We determined the percentage of infected cells (**A**) and the chlamydial inclusion size (**B**). In addition, we disrupted infected (MOI 0.15) wild type or *tlr4-*deficient T24/83 cells (5 × 10^5^ cells/well) 48 h post infection with glass beads and transferred the harvested elementary bodies to fresh wild type or *tlr4-*deficient T24/83 cell cultures (2.5 × 10^5^ cells/well). Twenty-four hours later, we determined the percentage of infected cells (**C**) and size (**D**) of chlamydial inclusions by fluorescence microscopy. The graphs depict the data of three independent experiments. ***, **** unpaired t test, *P* < 0.001, *P* < 0.0001, respectively.

**Fig. 11:**
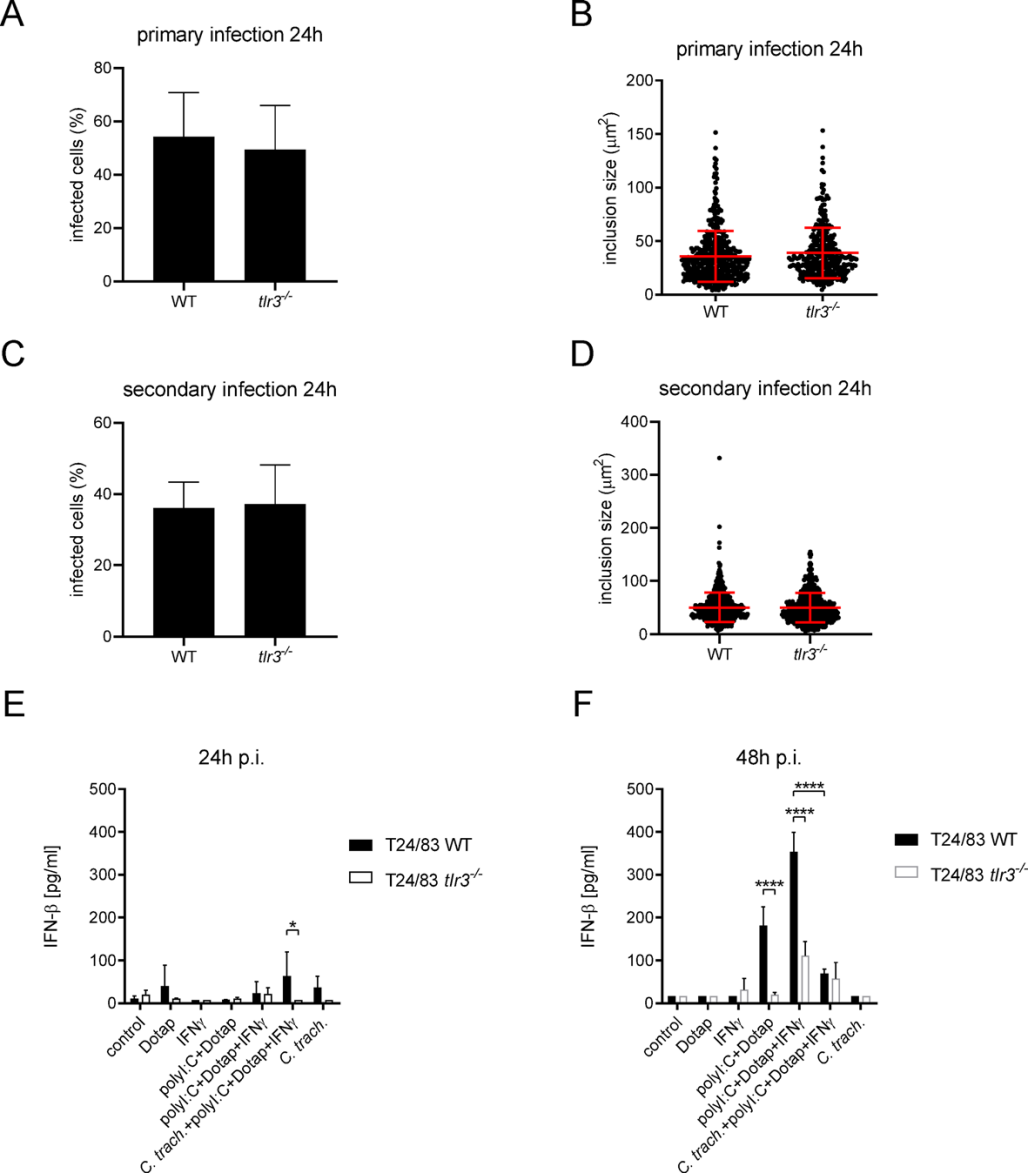
TLR3-dependent development of *C. trachomatis* serovar D inclusions. We infected wild type or *tlr3-*deficient T24/83 cells (2.5 × 10^5^ cells/well) with *C. trachomatis* serovar D (MOI 1) for 24 h. We fixed, permeabilized and stained the cells with an ACI-FITC anti-chlamydia antibody to detect chlamydial inclusions. We determined the percentage of infected cells (**A**) and the chlamydial inclusion size (**B**). In addition, we disrupted infected (MOI 0.15) wild type or *tlr3-*deficient T24/83 cells (5 × 10^5^ cells/well) 48 h post infection with glass beads and transferred the harvested elementary bodies to fresh wild type or *tlr3-*deficient T24/83 cell cultures (2.5 × 10^5^ cells/well). Twenty-four hours later, we determined the percentage of infected cells (**C**) and the size (**D**) of chlamydial inclusions by fluorescence microscopy. The graphs depict the data of three independent experiments. In (**E**, **F**) we infected WT or *tlr3*^*−/−*^ T24/83 cells with *C. trachomatis* serovar D (MOI 5) for 2 h. Subsequently, we stimulated the cells with IFN-γ (200 ng/ml) for 18 h and thereafter transfected the cells with polyI:C (5 µg/ml) plus dotap. We determined the amount of IFN-β in the culture supernatant by ELISA 24 h (**E**) and 48 h (**F**) post infection. The experiment was repeated twice with similar results. *, **** two way ANOVA post-hoc-Test Tukey, *P* < 0.05, *P* < 0.0001, respectively.

We then analyzed whether TLR3 or TLR4 influence the replication of the intracellular pathogen. We infected wild type and *tlr4-*deficient T24/83 cells with *C. trachomatis* serovar D and measured the number and the size of the chlamydial inclusions. The percentage of infected cells did not differ significantly (Fig. 10A) but the size of chlamydial inclusions was significantly smaller in *tlr4-*deficient cells compared to wild type T24/83 cells (Fig. 10B). Transfer of the chlamydial progeny to a second cell culture revealed that the percentage of infected cells and the size of chlamydial inclusions were significantly smaller (Fig. 10C, D). We then performed additional experiments to explore the relevance of TLR4 for the development of *C. trachomatis* serovar D inclusions during the first or second culture. Thus, we infected wild type T24/83 cells in the first culture and transferred the chlamydial progeny to TLR4-deficient T24/83 cells and vice versa. These experiments revealed that the number of *C. trachomatis* serovar D infected cells in *tlr4-*deficient or wild type T24/83 cells did not differ significantly while the size of chlamydial inclusions were at this time slightly larger in *tlr4-*deficient cells (Fig. S10). In summary, we think that TLR4 signaling may modulate chlamydial replication, but does not control it. *Tlr3-*deficiency in T24/83 cells also did not influence the percentage of infected cells nor the inclusion size during infection (Fig. 11A, B). TLR3 did also not influence chlamydial replication upon transfer of the progeny to a second cell culture (Fig. 11C, D). However, *C. trachomatis* serovar D impaired the TLR3-dependent polyI:C/doptap-induced release of IFN-β 48 h post infection (Fig. 11E, F). Taken together, it appears that neither TLR4 nor TLR3 are involved in the induction of an inflammatory response in *C. trachomatis* serovar D infected T24/83 cells. Furthermore, both TLRs exert no relevant influence on the replication of the pathogen.

## Discussion

Our results demonstrate that an infection with *C. trachomatis* serovar D triggered no detectable secretion of the pro-inflammatory cytokines TNF-α, IL-6, IL-8, IL-1β or IFN-β by the human bladder carcinoma cell line T24/83, although *tnf*, *il6*, and *il1β* were transcribed to some extent. T24/83 cells were principally able to secrete such cytokines as demonstrated by their release upon stimulation with polyI:C or LPS, well known TLR3/RIG-I/melanoma differentiation-associated gene 5 (MDA5) or TLR4 ligands, respectively, and by the infection with the uropathogenic *E. coli* strain CFT073. With the exception of IL-6, HeLa cells also did not secrete pro-inflammatory cytokines upon infection with *C. trachomatis* serovar D, nor did they respond to polyI:C or LPS. Thus, at least a subset of human epithelial cell lines responds to the obligate intracellular pathogen with a weak pro-inflammatory cytokine reaction, which is detectable on the transcriptional but not on the translational level. Also on the transcriptional level, the fold change values of pro-inflammatory mRNAs in *C. trachomatis* serovar D infected T24/83 cells were low compared to polyI:C- or LPS-stimulated cells. We thus think, that mRNA levels of pro-inflammatory genes induced upon infection with *C. trachomatis* serovar D result in protein levels below the lower limit of detection. Moreover, the set of most induced genes elicited in T24/83 cells by *C. trachomatis* serovar D infection or stimulation with polyI:C or LPS differed, also indicating that *C. trachomatis* serovar D triggered TLR4 or TLR3/RIG-I/MDA5 not at all or in a different way in T24/83 cells.

### Role of TLRs to recognize *C. trachomatis*

A role of TLRs in the recognition of *C. trachomatis* inclusions is suggested by the recruitment of TLRs such as TLR2 around the chlamydial inclusion as demonstrated previously. Thus, the transfection of yellow fluorescent protein (YFP)-tagged TLR2 into HEK293 cells and subsequent infection with *C. trachomatis* serovar L2 resulted in the accumulation of this TLR around the chlamydial inclusion^7^. In addition, co-transfection of HEK293 cells with TLR2 and MyD88 revealed that both molecules co-localize in these cells^7^. TLR4 played a minor role in that study. We failed to detect TLR2 in T24/83 cells (Fig. S4), while HeLa cells express this TLR^26^. We demonstrate here that neither endogenous TLR4 nor TLR3 influenced the number and the size of *C. trachomatis* serovar D inclusions in a stable and reproducible manner. A role of TLR4 to sense *C. trachomatis* was suggested by the finding that THP-1 cells responded to *C. trachomatis* serovars E or L2 infection upon retroviral transduction of TLR4/CD14/MD2^27^. However, endogenous levels of TLR4 in THP-1 cells did obviously not suffice to recognize the pathogens. Taken together it appears that if endogenous levels of TLRs are recruited to chlamydial inclusions, they fail to trigger a pro-inflammatory response, which is visible at the translational level.

We failed to detect TLR4 and TLR3 in T24/83 with commercially available antibodies. Nevertheless, it would be interesting to explore, if these TLRs, at endogenous expression levels, are recruited to the chlamydial inclusion and integrated into the inclusion membrane. Moreover, it is also unclear how TLRs reach the intracellular inclusion. Since *C. trachomatis* serovar L2 inclusions fuse with Golgi complex-derived sphingomyelin containing vesicles and TLR3 is usually transported through the Golgi complex to the endosomal compartment, possibly sphingomyelin-containing vesicles might also harbor TLR3 in their membrane^28,29^.

The functional relevance of TLR3 for the recognition of *C. trachomatis* serovar D was previously explored in the human oviduct cell line OE-E6/E7^11^. Using CRISPR genome editing to disrupt TLR3, infected OE-E6/E7 cells secreted cytokines like IL-6 and IL-8 in a partially TLR3-dependent manner^11^. However, certain inflammatory responses such as type III interferon IL-29 and IL-28 A and the chemokine CCL5 did not depend on TLR3 in OE-E6/E7 cells^11^. Thus, the results of this study differ at least partially to the ones reported here. It appears that epithelial cell lines derived from different tissues respond to a *C. trachomatis* serovar D infection in a different manner and because of that, the extent of inflammation might vary between different parts of the female urogenital tract.

While we did not observe a TLR3- or TLR4-dependent transcription of pro-inflammatory genes such as *tnf*, *il6*, *il1β*, *irak2*, *ifi44l*, or *ifit2* upon infection with *C. trachomatis* serovar D, we detected that TLR3 dampened transcription of *irak2*, *ifi44l* and *ifit2*, and TLR4 *tnf*, *il1β* and *irak2* significantly. This finding was unexpected and a similar observation was to our knowledge not reported. It would make sense for an obligatory intracellular pathogen to manipulate TLR-responses in such a way that inflammatory responses are dampened, however, mechanisms involved in this process are presently unclear.

Our results also demonstrated that TLR3 and TLR4 were functionally competent in T24/83 cells, as the uropathogenic *E. coli* strain CFT073 induced the secretion of TNF-α and IL-6 in a TLR3- and TLR4-dependent manner. This positive influence of TLR3 and TLR4 on cytokine secretion by CFT073 is in contrast to the negative influence of TLR3 and TLR4 on cytokine transcription induced by *C. trachomatis* serovar D. If *C. trachomatis* serovar D triggered these signal-competent TLRs in T24/83 cells, they had no influence on the replication of the pathogen.

We did not detect a significant activation of transcription factors NF-κB and IRF3 induced by *C. trachomatis* serovar D in T24/83 and HeLa cells 4 h post infection. Possibly, IκB degradation and IRF3 phosphorylation occurred at an earlier time point post infection with *C. trachomatis* serovar D. However, we consider this possibility as unlikely, since endotoxin and polyI:C triggered these signaling cascades 4 h post stimulation and they should activate the cells faster than an infection with *C. trachomatis* serovar D residing in a membrane-bound inclusion. A similar finding was reported for *C. trachomatis* serovar E LPS in comparison to *E. coli* LPS upon intraperitoneal injection of mice^30^. Additionally, NF-κB-reporter THP-1 cells failed to induce the transcription factor upon infection with *C. trachomatis* serovars E or L2^27^. These cells also failed to induce the NF-κB-reporter upon stimulation with pure monomeric endotoxin from *C. trachomatis* serovar E or L2^27^. Thus, not only the epithelial cells T24/83 and HeLa but also human monocytic THP-1 cells did not activate NF-κB upon infection with *C. trachomatis* serovars.

Consequently, we failed to detect secretion of cytokines by T24/83 and HeLa cells upon infection with *C. trachomatis* serovar D with the exception of IL-6 by infected HeLa cells, which we detected after an infection time of 40 h. In this case DAMPs released by infected and dying HeLa cells might have triggered IL-6 release. In contrast, T24/83 cells did not secrete cytokines even after 40 h of infection with *C. trachomatis* serovar D. Nevertheless, our microarrays revealed that transcription of cytokine genes such as *il1α*, *il1β*, *il6*, *il11* and *tnf* (Fig. 1A, D) 4 h p.i. and of interferon induced genes such as *ifit2* and *ifi44l* (Fig. 2A, D) 24 h p.i. was significantly induced. Reverse transcription real time PCR confirmed this observation for *il1β*, *il6*, *tnf* and *ifit2*, but not for *ifi44l* (Fig. 9). We did not evaluate *il1α* and *il11* by RT-PCR. However, the extent of gene transcription was obviously not sufficient to generate cytokines on the protein level above the detection threshold of our sensitive ELISA systems.

### Interference of PRR-signaling by *C. trachomatis* serovar D

As discussed above, the TLR3/RIG-I/MDA5 and TLR4 signaling cascades were functional in T24/83 cells as indicated by the secretion of IFN-β by polyI:C- and IL-6 by LPS-stimulation, respectively. Moreover, the cells recognized the uropathogenic *E. coli* strain CFT073 in a TLR3- and TLR4-dependent manner. The TLR4/RIG-I/MDA5 ligand induced responses were also not impaired by the simultaneous infection with *C. trachomatis* serovar D, although we infected the majority of cells. However, TLR3-induced responses were impeded by *C. trachomatis* serovar D infections. These findings indicate that *C. trachomatis* serovar D impaired pattern-recognition receptors in a very selective manner. Partially similar findings were observed in a human fallopian tube epithelial cell culture model^31^. Accordingly, infection of these epithelial cells with *C. trachomatis* serovar E resulted in downregulated protein expression of TLR-signaling and immune signaling, while several chemokines such as CXCL10, CXC11 and CCL5 were detected in infected fallopian tube epithelial cells^31^.

### Influence of TLRs on chlamydial replication

Unexpectedly, we observed that neither TLR3 nor TLR4 influenced the development of chlamydial inclusions of *C. trachomatis* serovar D in the uroepithelial cell line T24/83. The role of TLR3 for the replication of *C. trachomatis* serovar D was also analyzed in the human oviduct cell line OE-E6/E7^11^. Authors demonstrated that chlamydial inclusion were larger and were aberrantly shaped in *tlr3-*disrupted OE-E6/E7 cells and transfer of the cell lysate to HeLa cells revealed a significantly higher chlamydial number^11^. These rather contrasting findings remain at present unexplained. Interestingly, *C. trachomatis* serovar D infected *tlr3-*deficient OE-E6/E7 cells were able to transfer the bacterium for much longer periods than wild type cells, as if the chlamydial inclusion is more stable in the absence of TLR3. In both studies, epithelial cells were lysed to transfer *Chlamydia* to fresh cells. It would therefore be interesting to analyze whether TLR4 and TLR3 would influence the spontaneous release of newly generated EBs from infected epithelial cells.

### Pattern of gene expression induced by *C. trachomatis*.

*C. trachomatis* serovar D induced the genes matrix metalloproteinases 1 (MMP1) and 3 (MMP3) most strongly in contrast to endotoxin and polyI: C (Fig. 6). Similarly, Xu et al. also reported that *C. trachomatis* serovar L2 induced MMP1, 2, 3 TLR3-dependently in the human oviduct cell line OE-E6/E7^11^. In case of *C. trachomatis* serovar D infection, MMP2 and 10 were TLR3-dependently produced^11^. MMPs play a role in tissue remodeling induced by *C. muridarum* since treatment of mice with an unselective MMP-inhibitor prevented an ascension of the infection and the chronic inflammatory consequences^32^. More specifically, MMP9 fosters the pathogenesis of urogenital *C. muridarum* infection in mice as MMP9-deficient mice showed a reduced formation of hydrosalpinx^33^. It thus appears that *C. trachomatis* serovars induce preferentially genes for tissue remodeling than for inflammation.

A limitation of our study is the use of the human bladder cancer cell line T24/83, which replicates *C. trachomatis* serovar D without restriction, but does not represent a genital epithelial cell. To generalize our findings, future experiments need to perform similar experiments as presented here with immortalized or primary cells of the human genital tract. In summary, we show here that the infection of the human uroepithelial cell line T24/83 with *C. trachomatis* serovar D triggers only a weak transcriptional pro-inflammatory response, which is insufficient for the secretion of pro-inflammatory cytokines. Furthermore, we find no evidence that TLR4 or TLR3 influence the replication of *C. trachomatis* serovar D infection in T24/83 cells in contrast to other epithelial cells. The rather low pro-inflammatory potential of *C. trachomatis* serovar D shown in this study may help to explain the often asymptomatic progression of genital *Chlamydia trachomatis* serovar D-K infections.

## Supplementary Information

Below is the link to the electronic supplementary material.

Supplementary Material 1

Supplementary Material 2

## Data Availability

The microarray data generated and analyzed during the current study are available in the Gene Expression Omnibus repository (https://www.ncbi.nlm.nih.gov/geo/), accession number GSE153235. All other data are available on request from the authors. Please contact SK or TM.
