# Supplementary material for: Chlamydia trachomatis serovar D replicates in uroepithelial T24/83 cells in the absence of overt inflammation
Source: Sci Rep. 2026 Jul 20;16:22710. doi: 10.1038/s41598-026-62091-4 (PMC13385356; doi:10.1038/s41598-026-62091-4)
Supplement: Supplementary file 1 — Supplementary Material 1 [file 41598_2026_62091_MOESM1_ESM.docx]

**Figure S1.** Potential of uroepithelial cell lines and HeLa cells to secrete IFN-β. We cultured uroepithelial cells lines UMUC-3, RT112, UROtsa, T24/83 and HeLa-cells (60.000 cells/well, 24-well plate) for 4h (A, B, C) or 6h (D, E). Cells were either not treated (control), treated with Lipofectamine 3000 (3 μl/ml) or Lipofectamine 3000 (3 μl/ml) plus polyI:C (5 μg/ml). Subsequently, we determined the IFN-β concentration in the culture supernatant by ELISA. Cultures were performed in triplicates. We performed the experiments once (A, B, C) or thrice (D, E). One representative experiment is shown (D, E). *one way ANOVA post-hoc test Tukey, *P*<0.05

**Figure S2.** *C. trachomatis* serovar D impairs polyI:C/dotap mediated release of IFN-β. T24/83 cells were infected with *C. trachomatis* serovar D (MOI 5) for 2h. Subsequently, we stimulated the cells with IFN-γ (200 ng/ml) for 18h and thereafter transfected the cells with polyI:C (5 µg/ml) plus dotap. We determined the amount of IFN-β in the culture supernatant by ELISA 24h (A) and 48h (B) post infection. Cultures were performed in triplicates. The experiment was repeated once with similar results. *One way ANOVA post-hoc test Tukey, **** *P*<0.0001

**
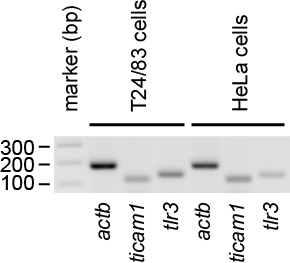
**

**Figure S3.** T24/83 and HeLa cells express *tlr3* and *ticam1*. Expression of *tlr3* and *ticam1* was analyzed by reverse transcription PCR. RNA was isolated using the RNeasy Mini Kit (Qiagen, Hilden, Germany). We used the Revert Aid First Strand cDNA Synthesis Kit (Thermo Fisher, Waltham, Massachusetts, USA) for reverse transcription. We used the primers tlr3_forward_ (5’-GGAAAGGCTAGCAGTCAT-3’) and tlr3_reverse_ (5’-CAGCAACTTCATGGCTAAC-3’), ticam1_forward_ (5’-GAGCCTGAGGAGATGAG-3’) and ticam1_reverse_ (5’-CAGTGGAGGTTGCATCTG-3’), actin_forward_ (5’-GAGCTACGAGCTGCCTGA-3’) and actin_reverse_ (5’-CACTGTGTTGGCGTACAG-3’) to amplify *tlr3*, *ticam1* and *actb*, respectively.


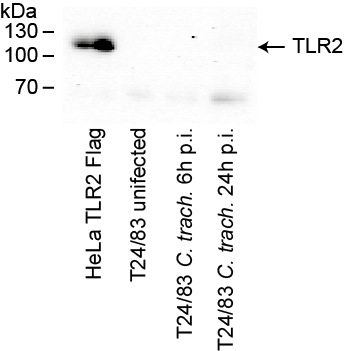


**Figure S4.** Expression of TLR2 in T24/83 cells. T24/83 cells were infected with *C. trachomatis* serovar D (MOI 1) for 6 or 24 hours or were not infected as indicated in the graph. HeLa cells transfected with the plasmid pFlag-hTLR2-CMV1 (kindly provided by Dr. Andreas Klos, Hannover, Germany) encoding a Flag-tagged TLR2 served as positive control. Cells were lysed with RIPA-buffer and TLR2 was detected by Western blotting using a TLR2-specific antibody (ab24192, abcam).

**
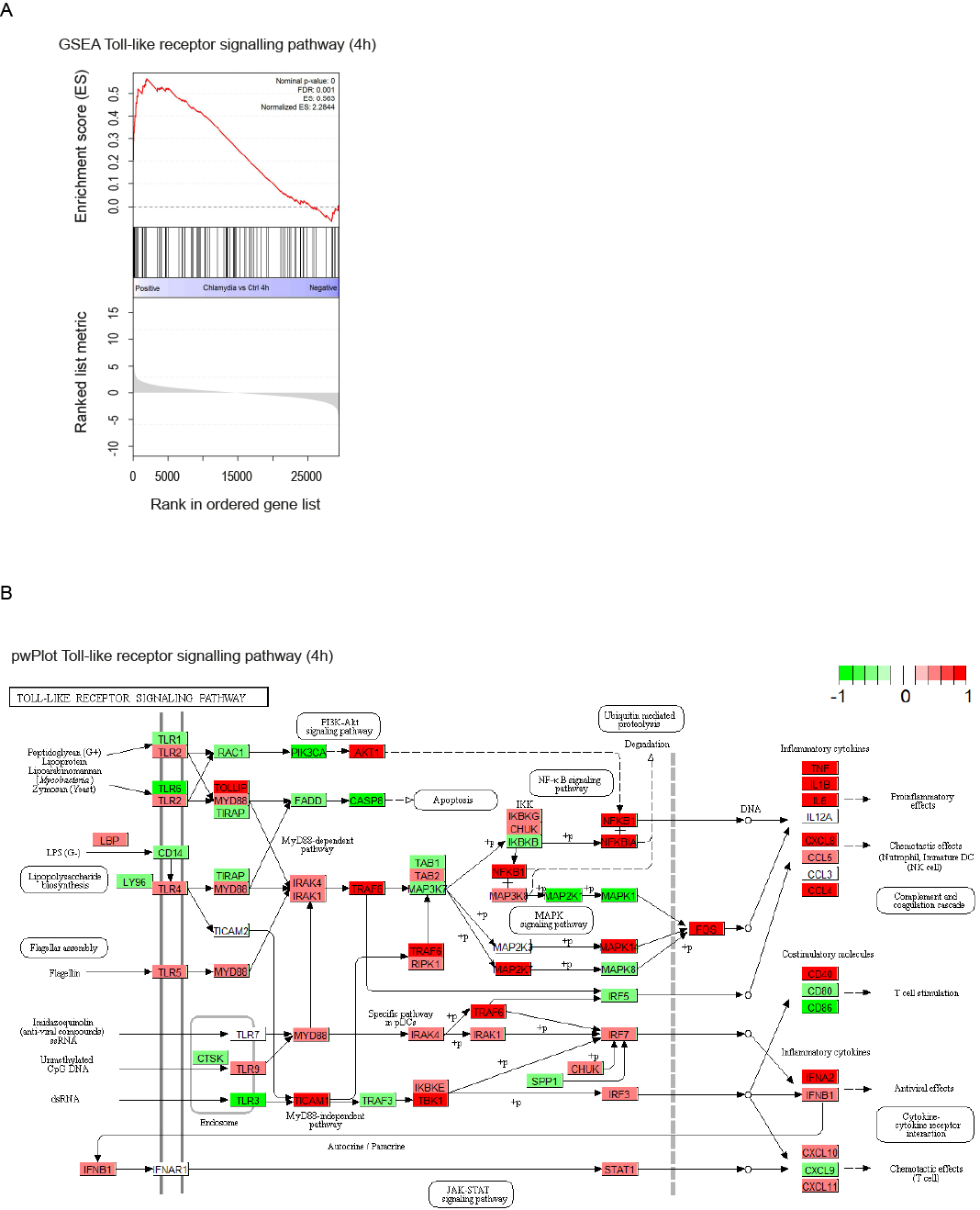
**

**Figure S5.** We infected T24/83 cells (5x10^5^ cells/well) with *C. trachomatis* serovar D (MOI 5) for 4h and analyzed their transcriptome by microarray. Graphs depict gene set enrichment analysis of Toll-like receptor signaling (A) and a pathway plot of the Toll-like receptor signaling pathway to show the regulation of individual genes (B). **
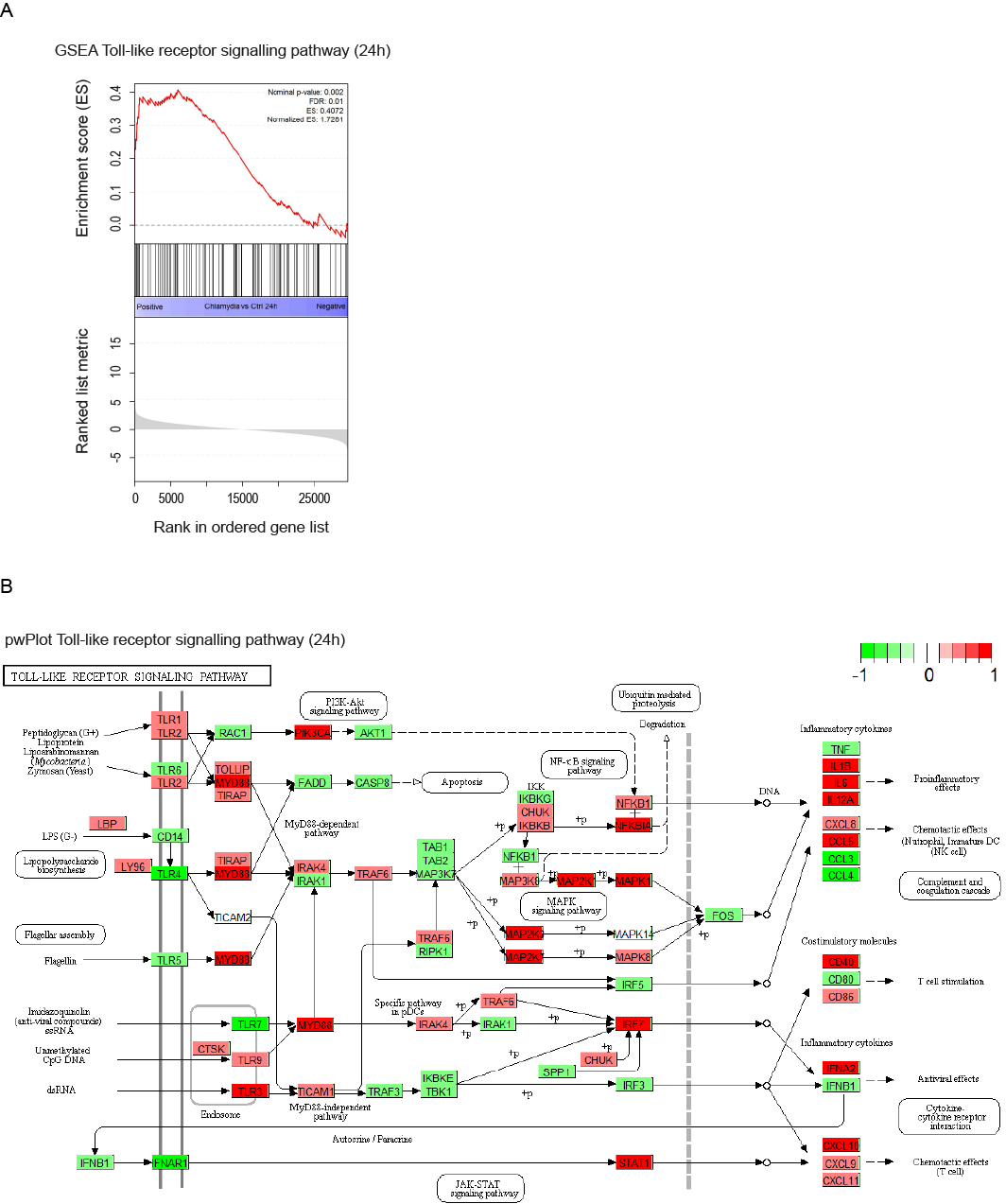
**

**Figure S6.** We infected T24/83 cells (5x10^5^ cells/well) with *C. trachomatis* serovar D (MOI 5) for 24h and analyzed their transcriptome by microarray. Graphs depict gene set enrichment analysis of Toll-like receptor signaling (A) and a pathway plot of the Toll-like receptor signaling pathway to show the regulation of individual genes (B).


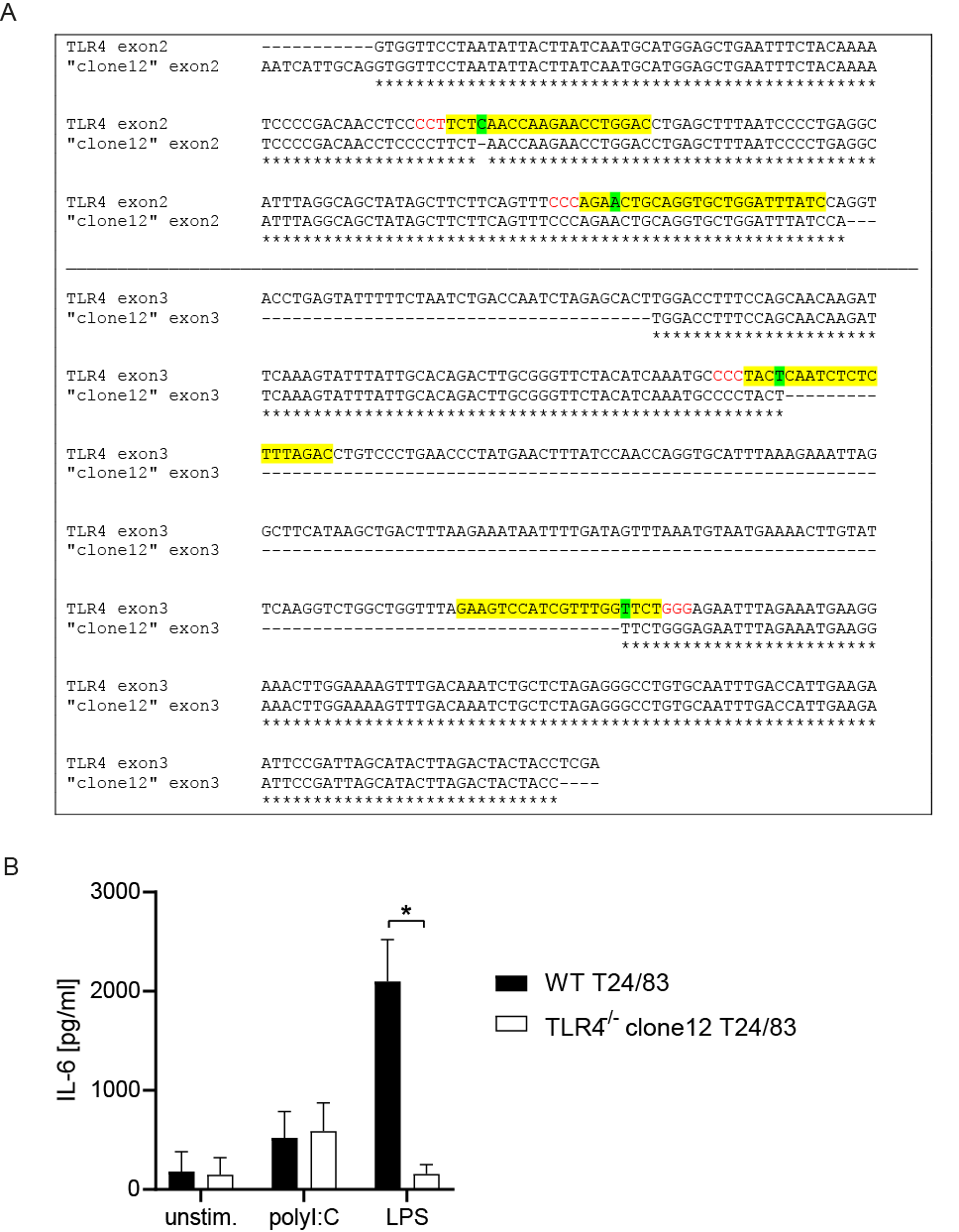


**Figure S7.** CLUSTAL Sequence Alignment results (MUSCLE) of exons 2 and 3 of T24/83 *tlr4^-/-^* clone12 with *tlr4* gene. (A) Sequence: asterisks signify complementary nucleotides; gRNA binding regions highlighted in yellow with green highlights indicating predicted CRISPR-Cas9 cutting sites; corresponding PAMs shown in red. The point deletion in exon 2 leads to a stop codon. (B) We stimulated wild type and *tlr4^-/-^* T24/83 clone12 with polyI:C (5 µg/ml) or LPS (1 μg/ml) and analyzed secreted IL-6 in the culture supernatant by ELISA. The graph represents three independent experiments. Each experiment was performed in triplicates. *one way ANOVA, post hoc test Tukey, *P*<0.05.

**
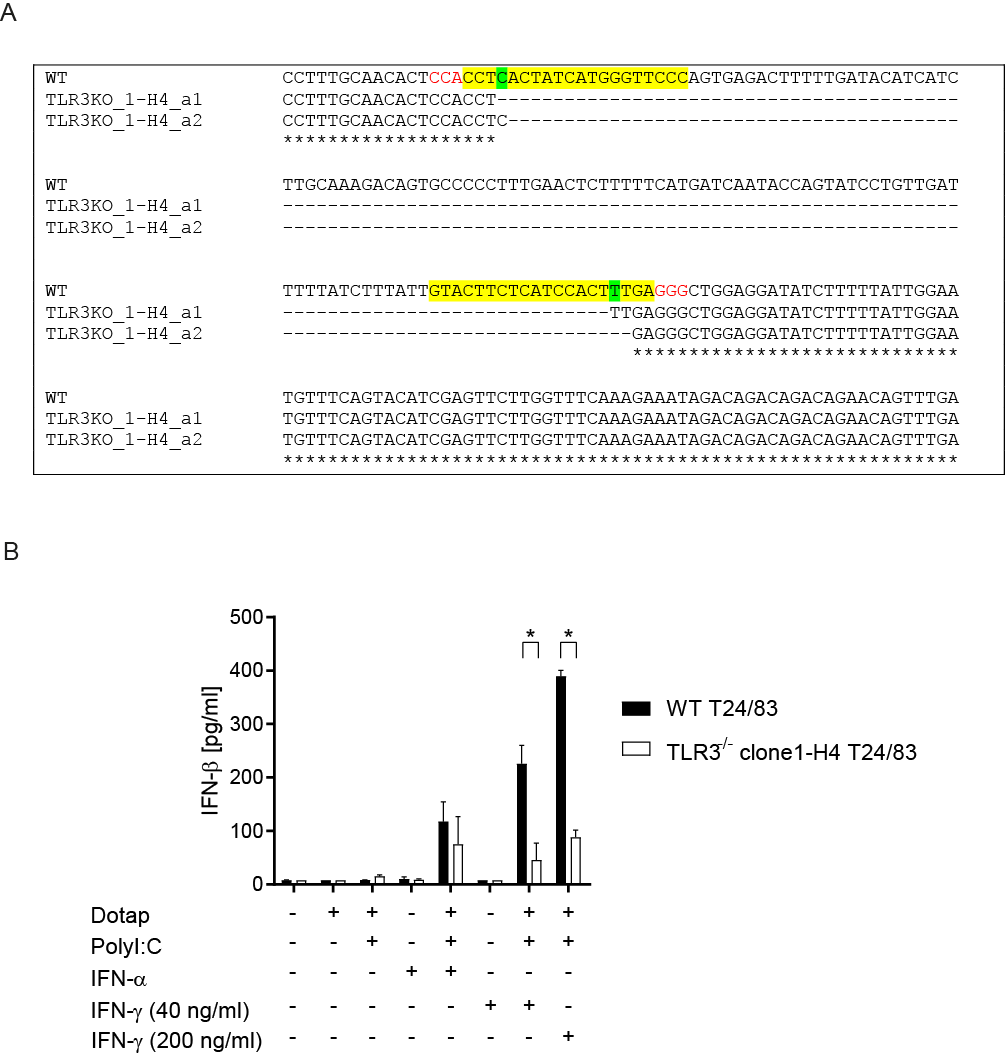
**

**Figure S8.** CLUSTAL Sequence Alignment results (MUSCLE) of exon 4 of T24/83 *tlr3^-/-^* clone1-H4 with *tlr3* gene. (A) Sequence: asterisks signify complementary nucleotides; gRNA binding regions highlighted in yellow with green highlights indicating predicted CRISPR-Cas9 cutting sites; corresponding PAMs shown in red. Deletions in exon 4 lead to downstream stop codons. (B) We transfected wild type and *tlr3^-/-^* T24/83 clone1-H4 with polyI:C/dotap (5 µg/ml), added IFN-α (1000 IU/ml) or IFN-γ as indicated and analyzed secreted IFN-β in the culture supernatant by ELISA. In addition to the depicted experiment, we repeated the experiment once with identical results. Each experiment was performed in triplicates. *two way ANOVA, post hoc test Tukey, *P*<0.05.

**Figure S9.** Influence of TLR3 and TLR4 on the transcription of pro-inflammatory genes post infection with *C. trachomatis* serovar D

We infected wild type, *tlr3*- or *tlr4*-deficient T24/83 cells (5x10^5^ cells/well) with *C. trachomatis* serovar D (MOI 3) for 4 or 24h as indicated in the graphs. We used uninfected cells as controls. We isolated the cellular RNA and performed a reverse transcription real time PCR. We used the primers listed in Tab. 2 to detect *tnf* (A), *il6* (B), *il1β* (C), *irak2* (D), *ifi44l* (E, F) and *ifit2* (G) mRNAs. We calculated the relative fold gene expression of samples as described in methods. Bars in each graph depicts the mean ± standard deviation of three independent experiments, each experiment was performed with three technical replicates.

**Figure S10.** Influence of TLR4 on the replication of *C. trachomatis* serovar D

We infected wild type or *tlr4*-deficient T24/83 cells (3x10^5^ cells/well) with *C. trachomatis* serovar D (MOI 0.15) for 48h, then disrupted the cells with glass beads and transferred the harvested elementary bodies to fresh wild type or *tlr4*-deficient T24/83 cell cultures (1.5x10^5^ cells/well) as indicated. Twenty-four hours later, we determined the number (A) and the size (B) of chlamydial inclusions by fluorescence microscopy. *, ** one way Anova, post hoc test Dunnett, *P*<0.05, *P*<0.01, respectively.
