# Supplementary material for: Chlamydia trachomatis serovar D replicates in uroepithelial T24/83 cells in the absence of overt inflammation
Source: Sci Rep. 2026 Jul 20;16:22710. doi: 10.1038/s41598-026-62091-4 (PMC13385356; doi:10.1038/s41598-026-62091-4)

Figure 3A Vinculin exposure time 20 min.

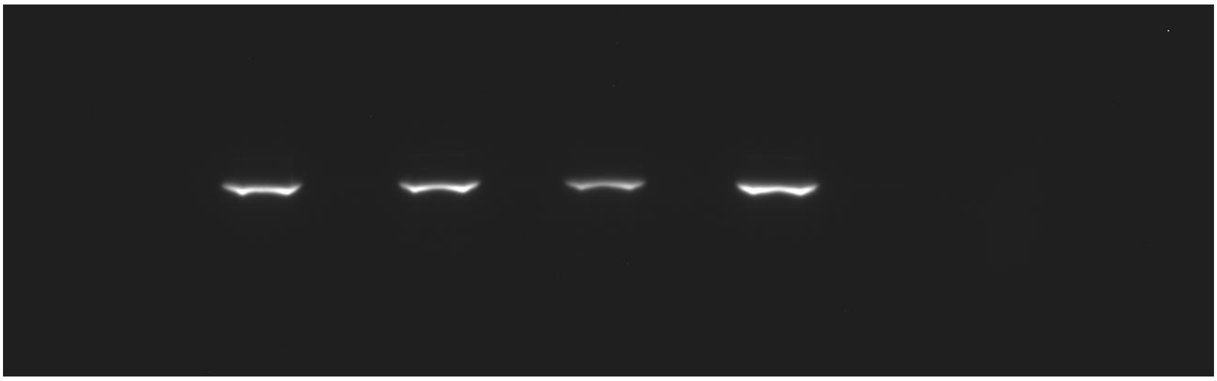

Figure 3A Marker for vinculin blot

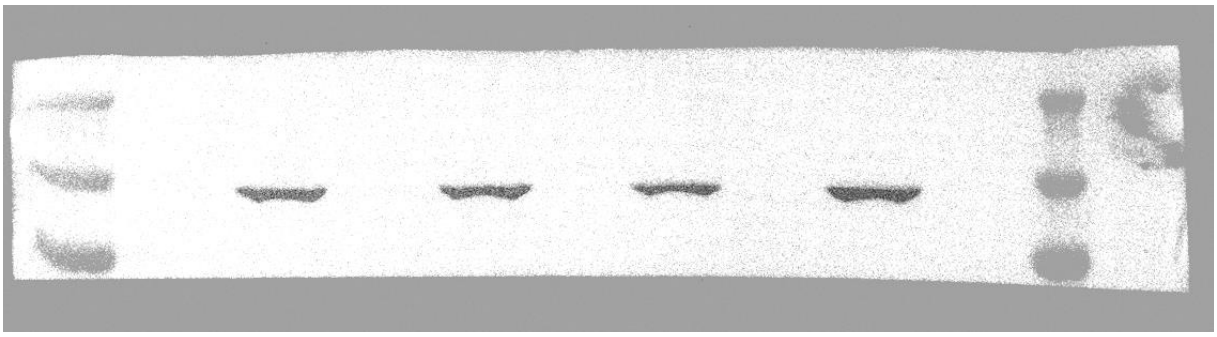

Figure 3A TBK1 exposure time 20 min.

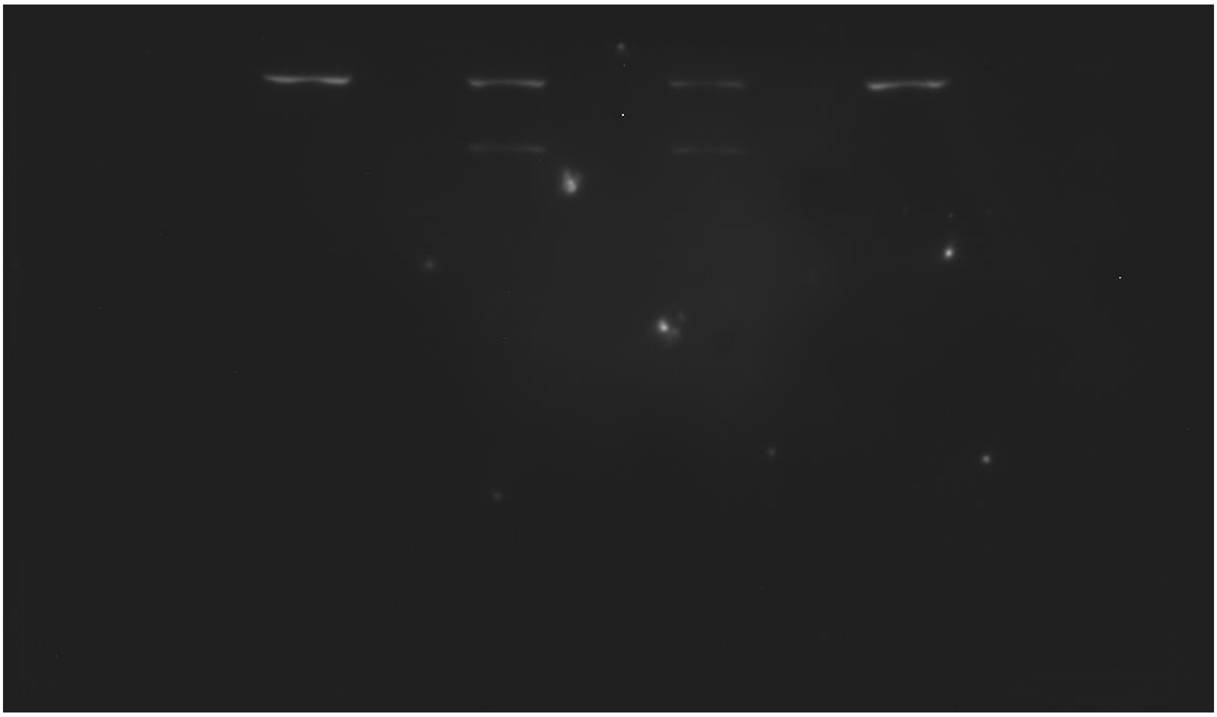

Figure 3A Marker for TBK1 blot

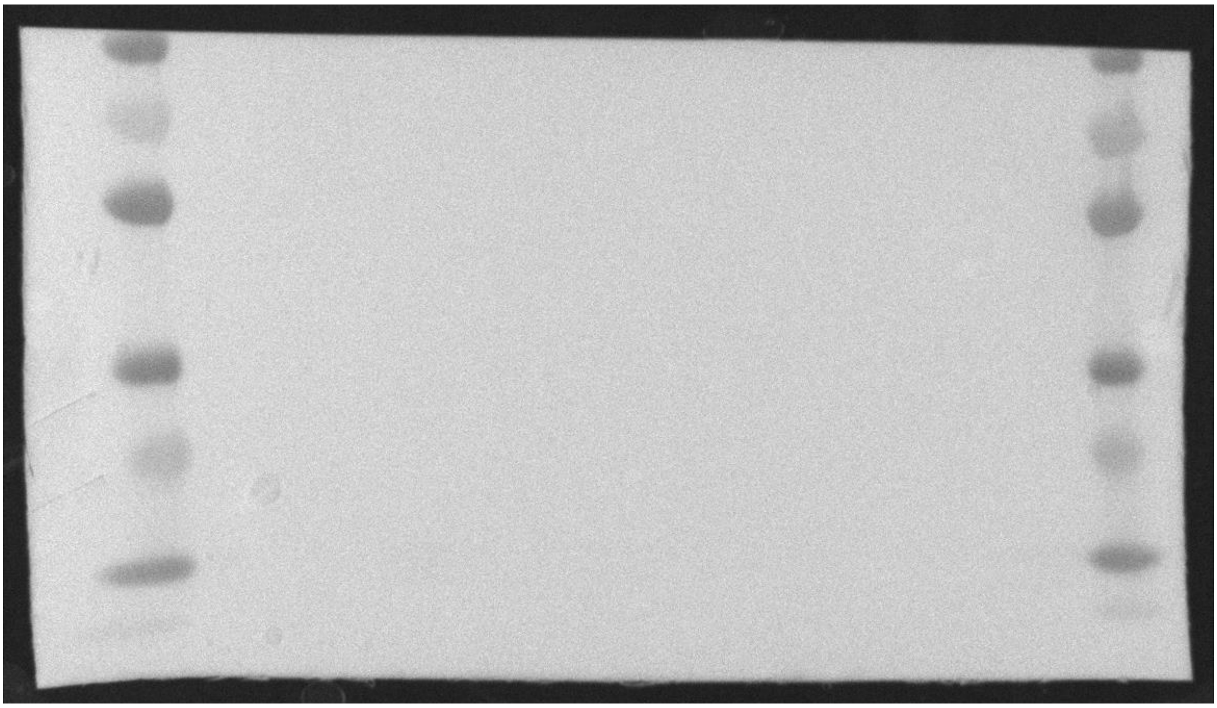

Figure 3A IRF3 exposure time 20 min.

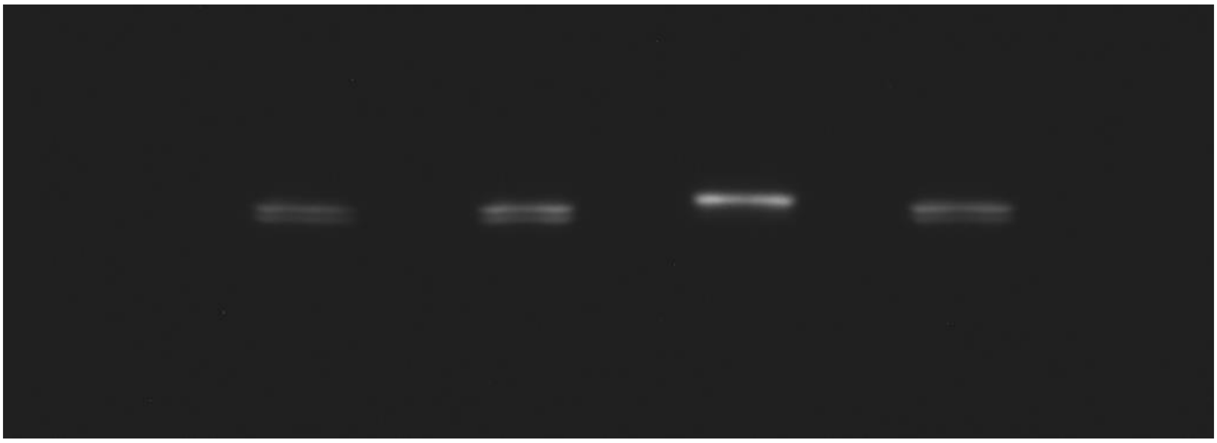

Figure 3A Marker for IRF3 blot

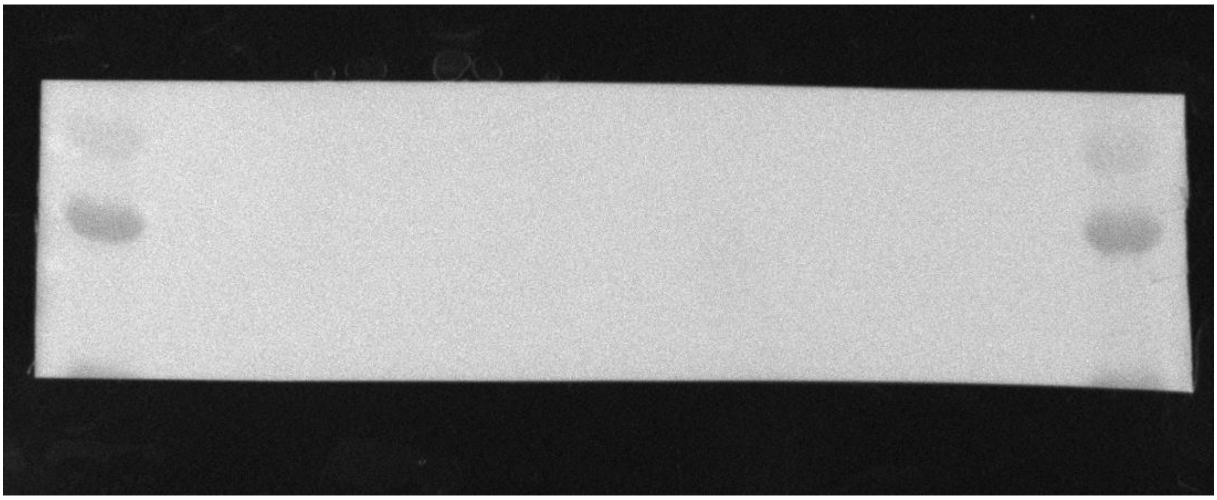

Figure 3A pIRF3(Ser386) exposure time 20 min.

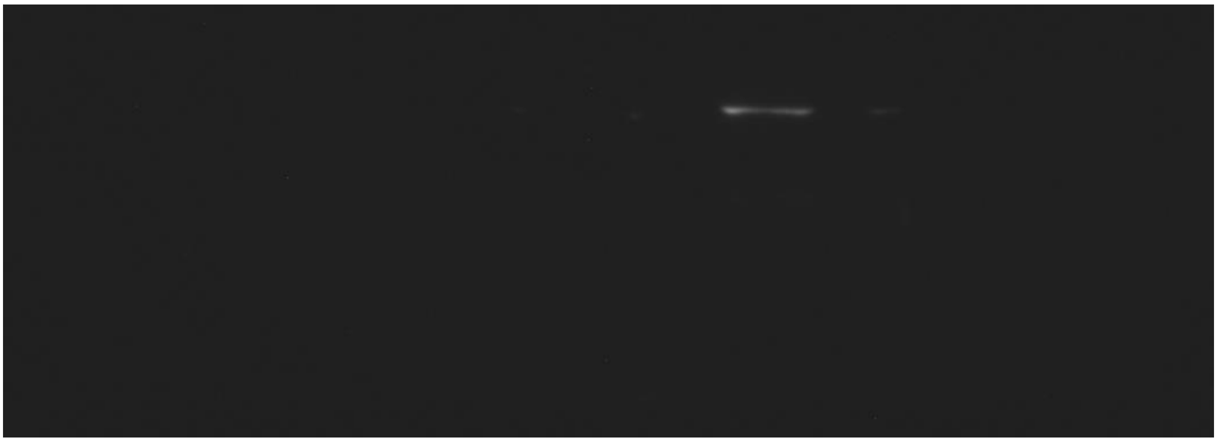

Figure 3A marker for pIRF3(Ser386) blot

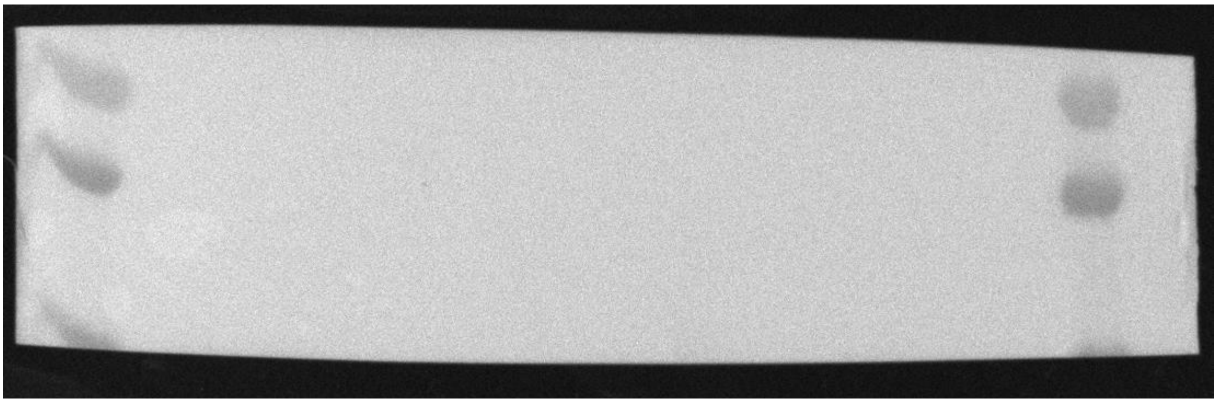

Figure 3A pIRF3(Ser396) exposure time 20 min.

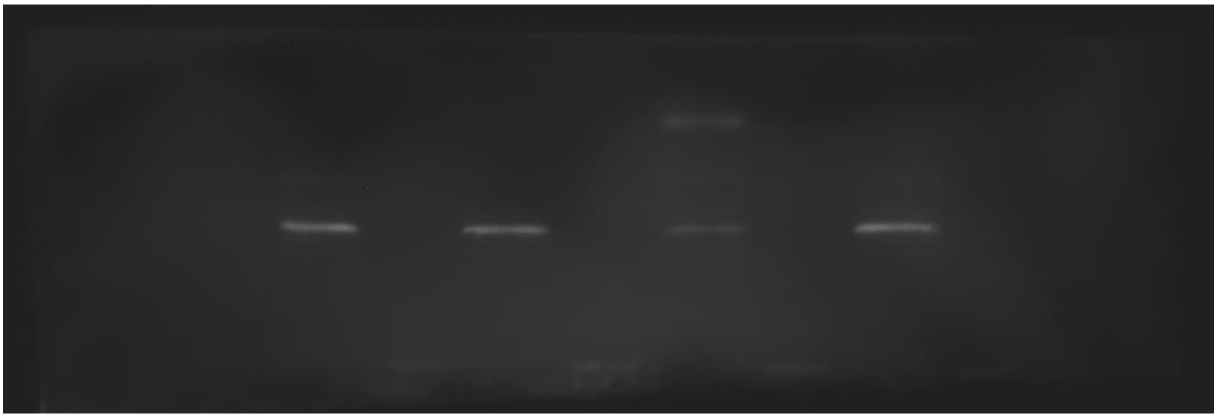

Figure 3A marker for pIRF3(Ser396) blot

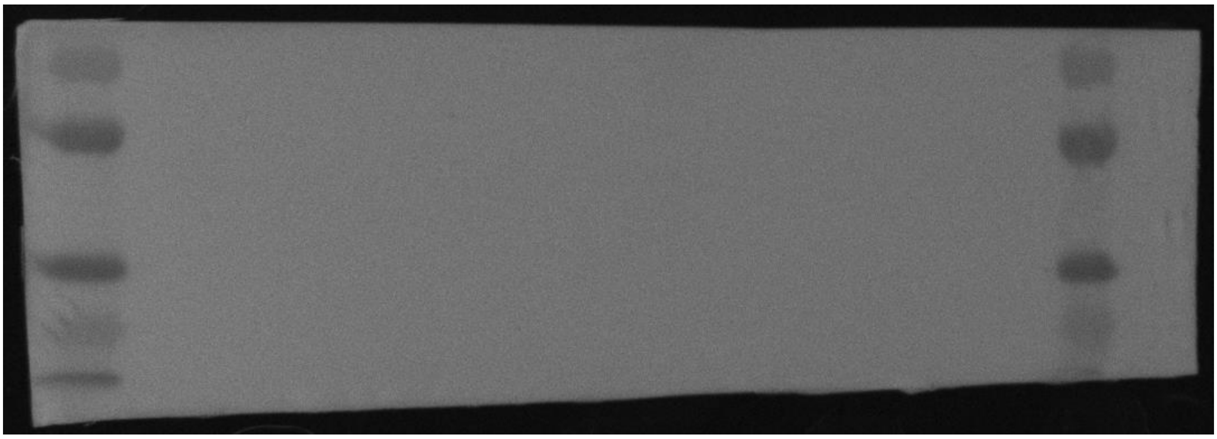

Figure 3A Histone exposure time 40s

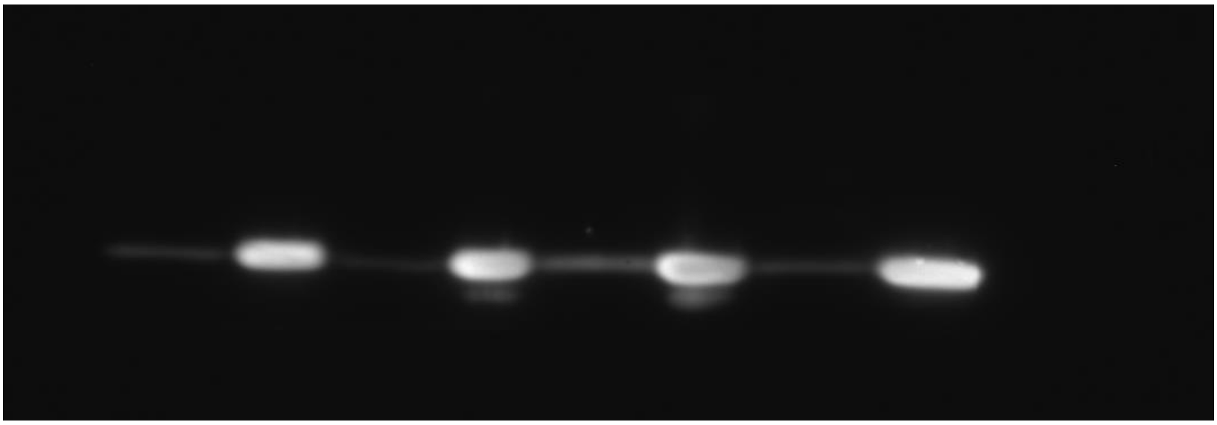

Figure 3A marker for Histone blot

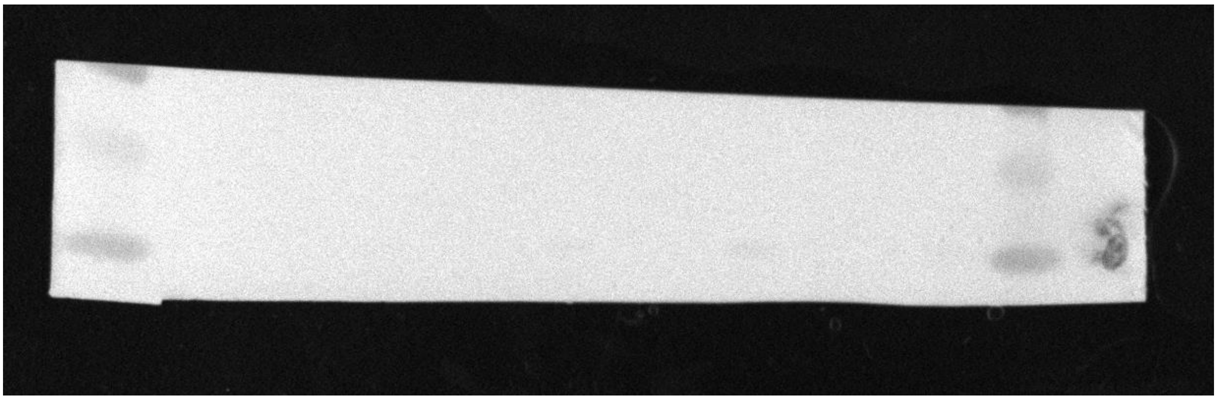

Figure 3B Vinculin exposure time 12 min.

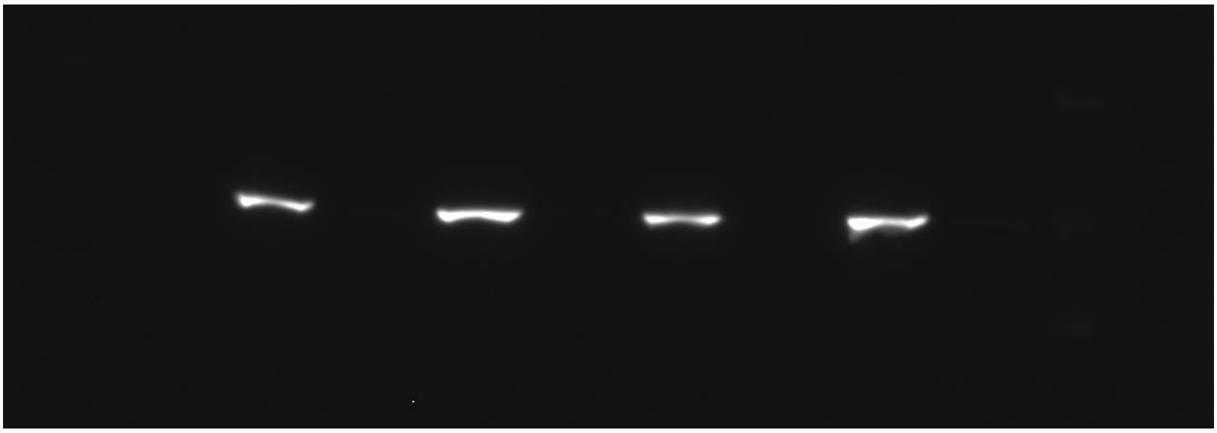

Figure 3B Marker for vinculin blot

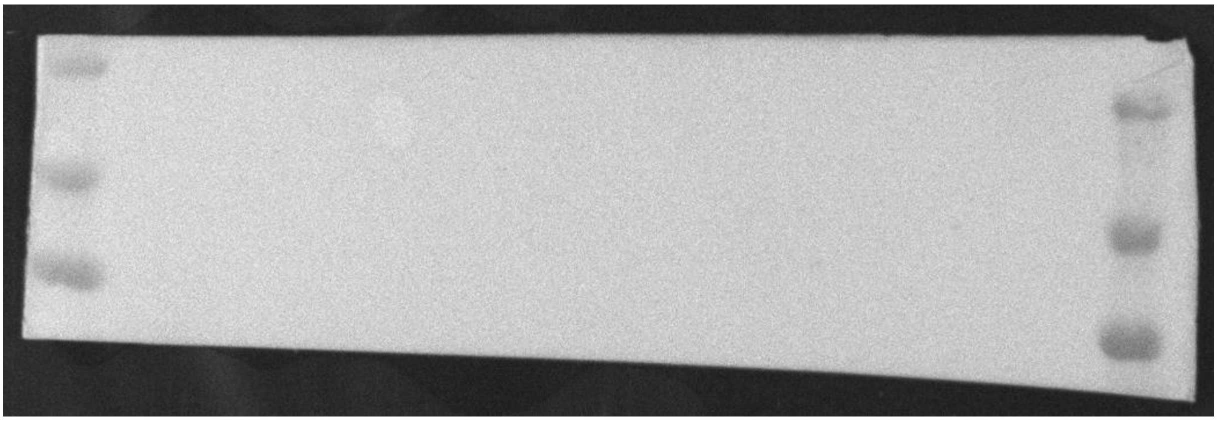

Figure 3B TBK1 exposure time 20 min.

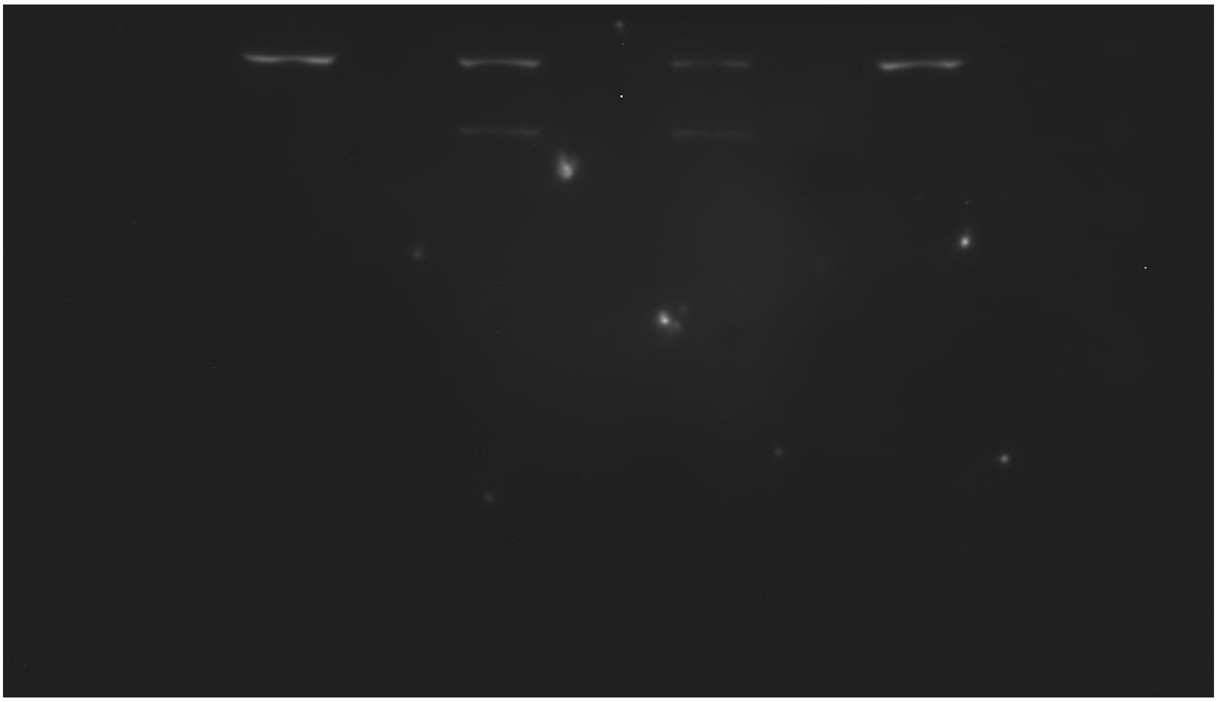

Figure 3B Marker for TBK1 blot

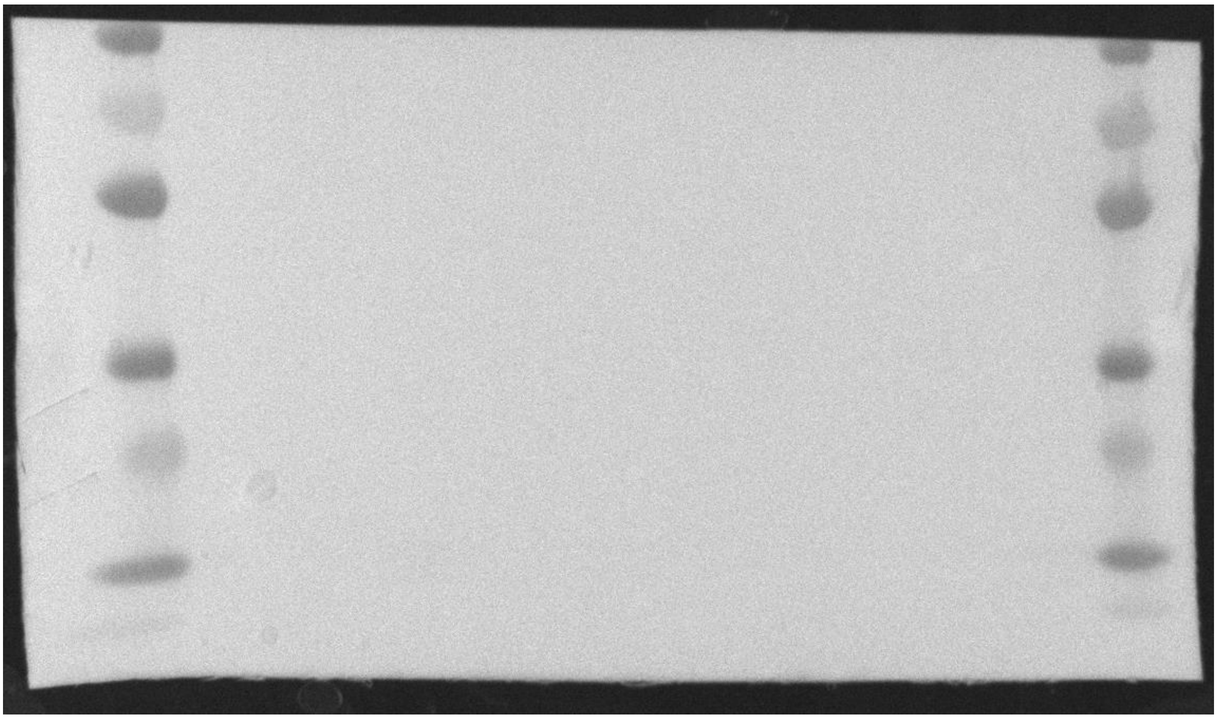

Figure 3B IRF3 exposure time 20 min.

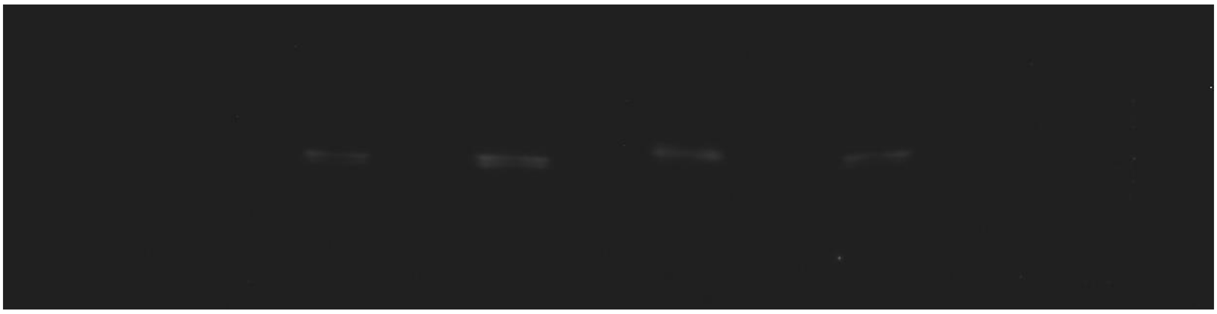

Figure 3B Marker for IRF3 blot

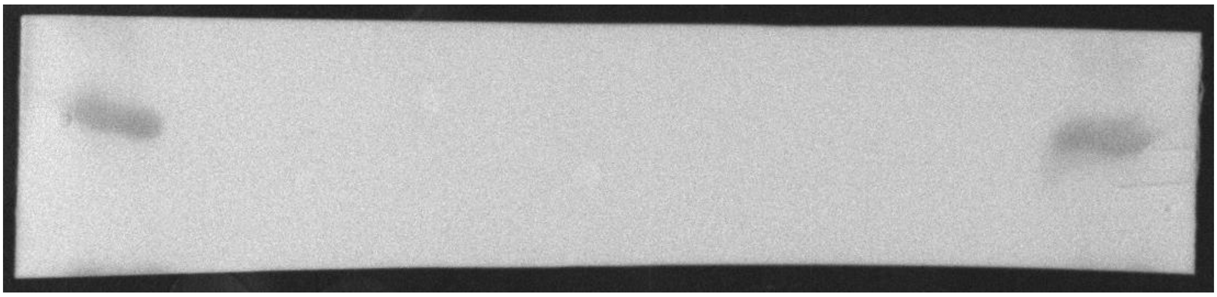

Figure 3B pIRF3(Ser386) exposure time 20 min.

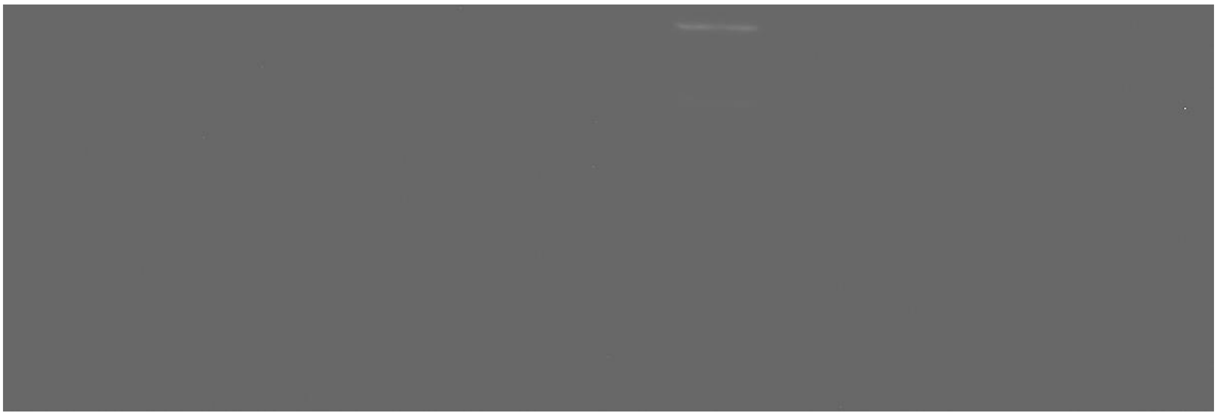

Figure 3B Marker for pIRF3(Ser386) blot

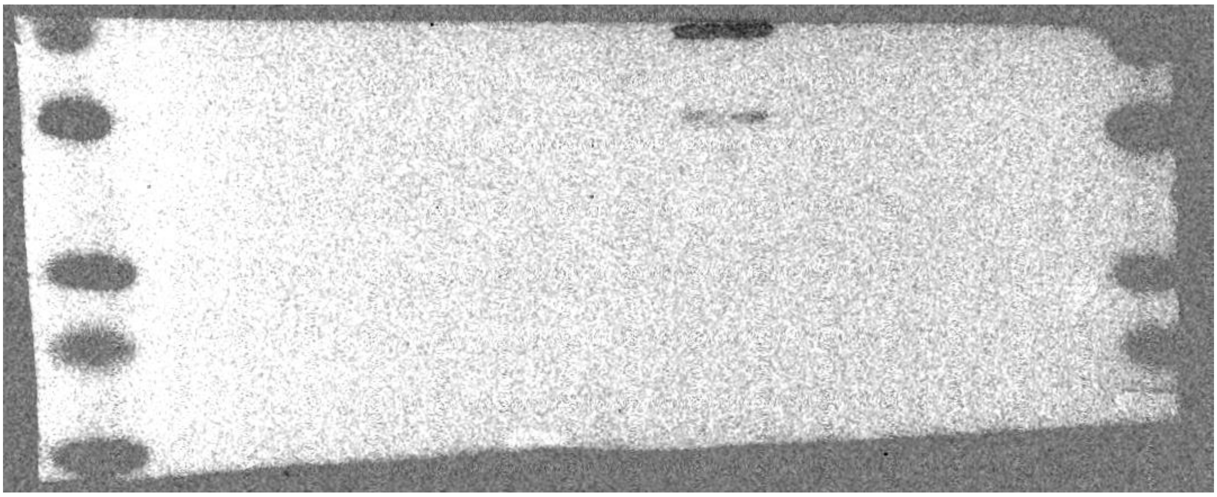

Figure 3B pIRF3(Ser396) exposure time 20 min.

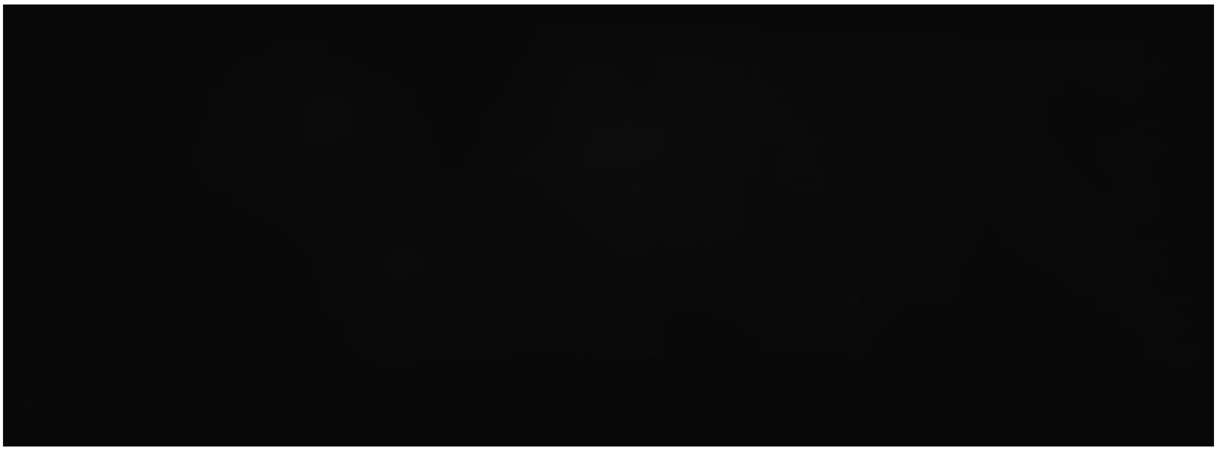

Figure 3B Marker for pIRF3(Ser396) blot

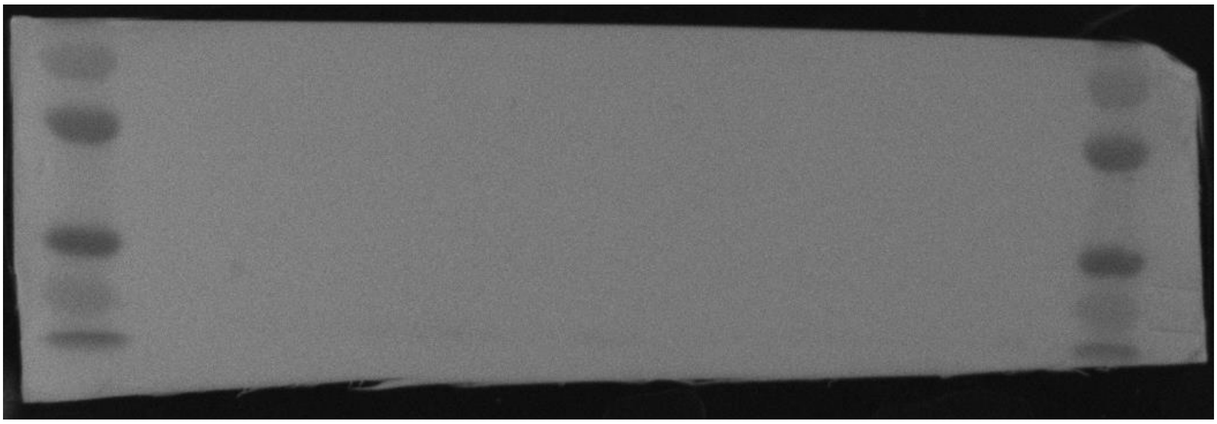

Figure 3B Histon exposure time 20s

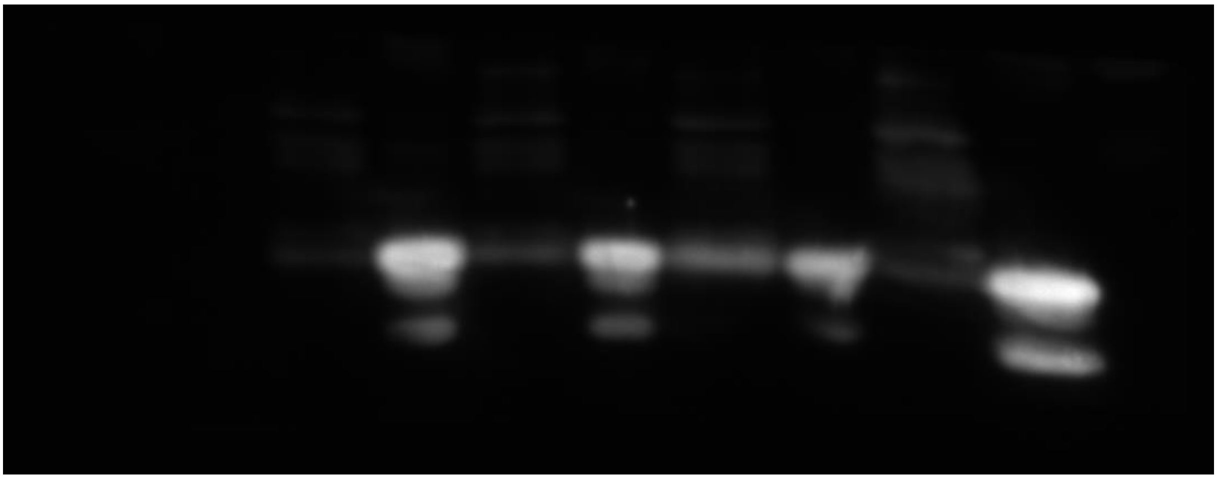

Figure 3B Marker for Histon blot

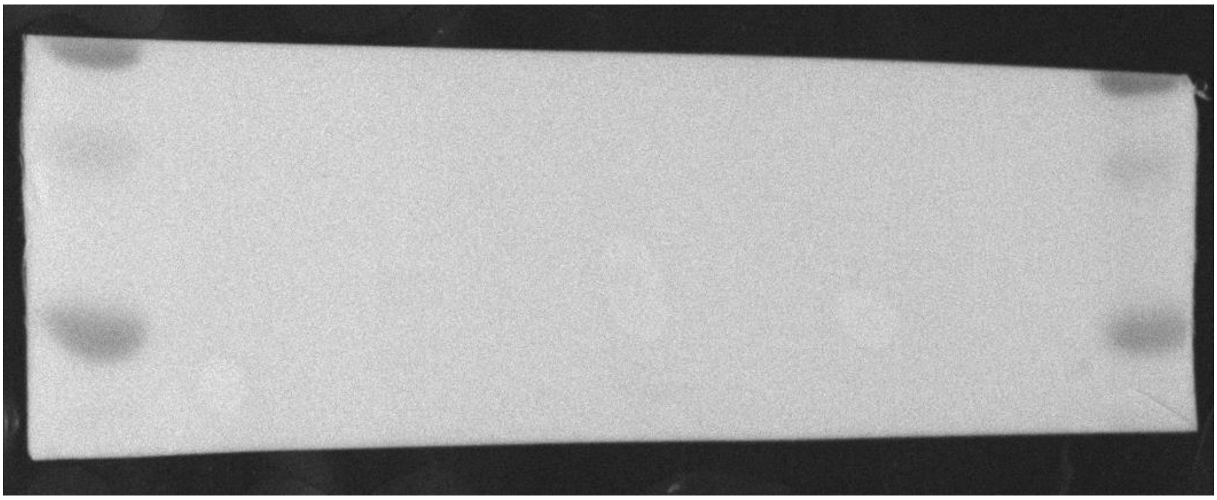

Figure 3C IκB exposure time 40 min.

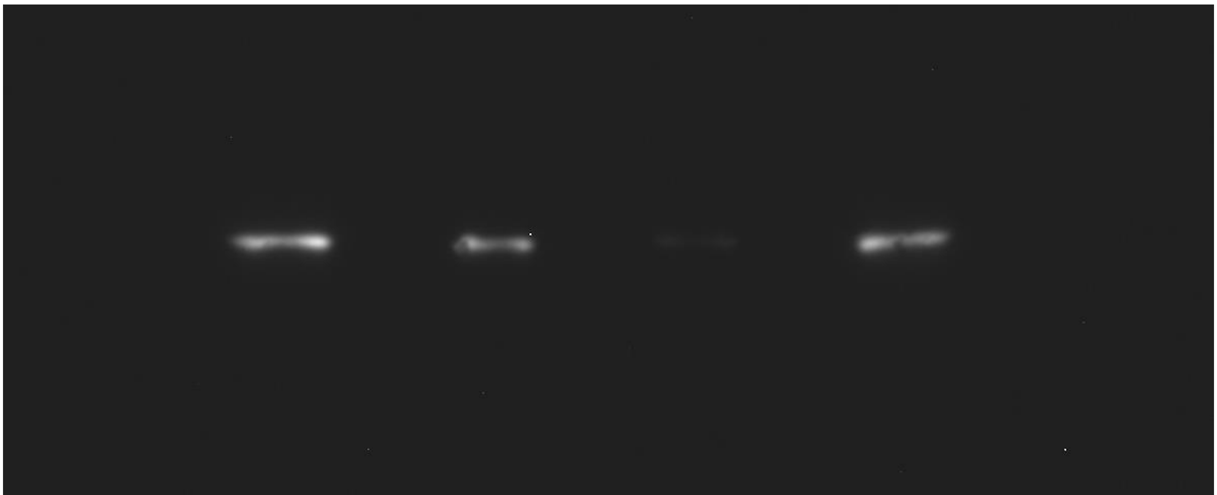

Figure 3C Marker for IκB blot

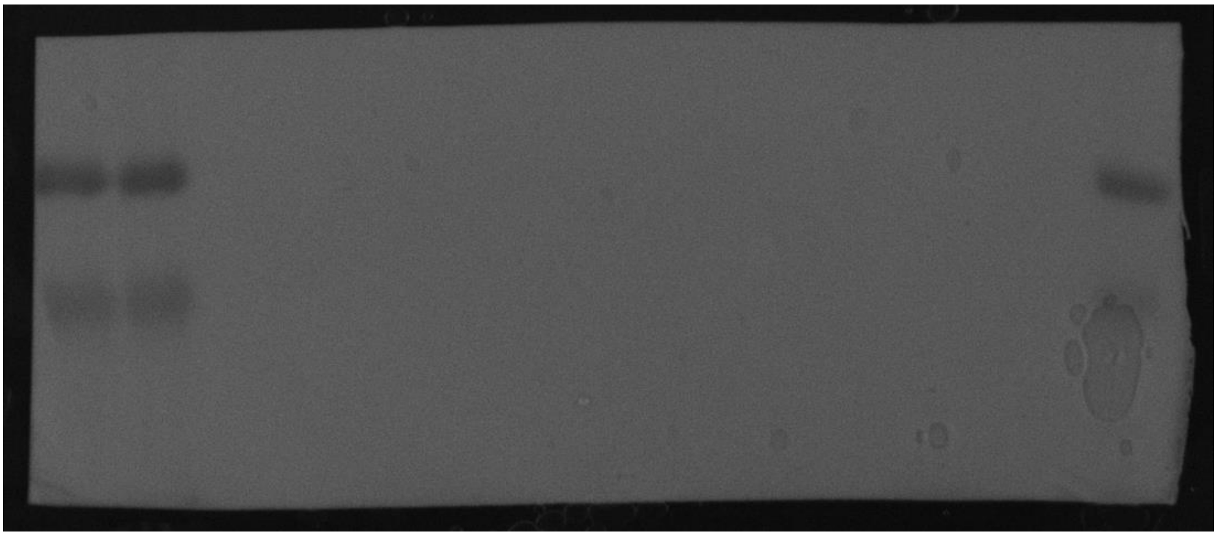

Figure 3C Vinculin exposure time 8 min.

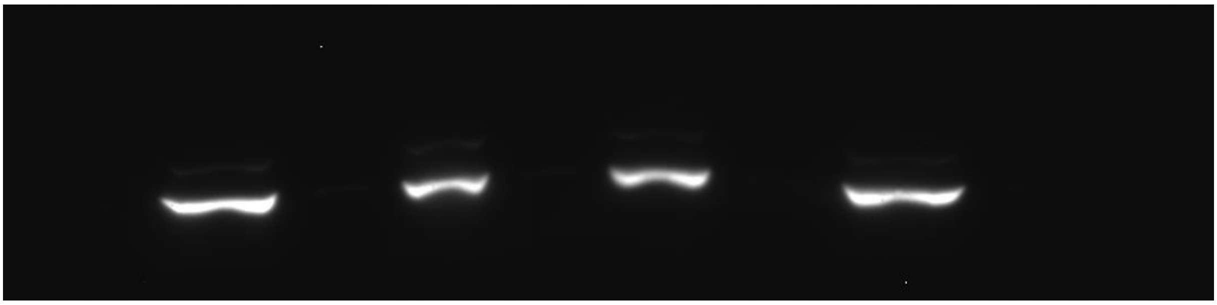

Figure 3C Marker for Vinculin blot

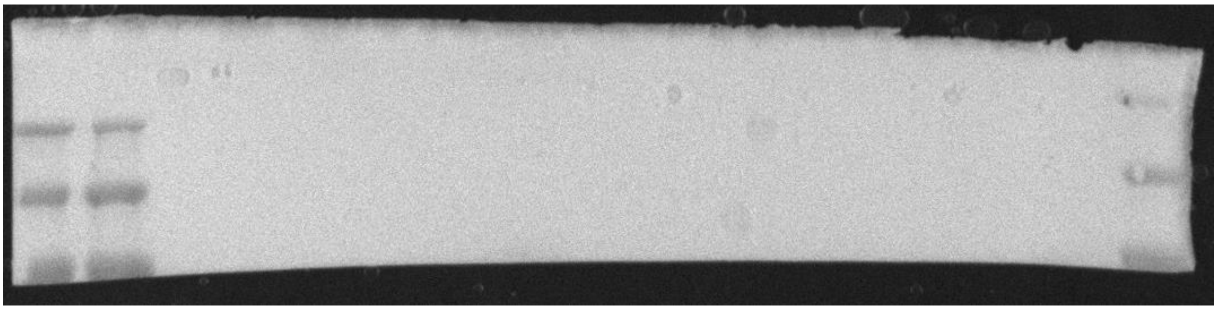

Figure 3D IκB exposure time 40 min.

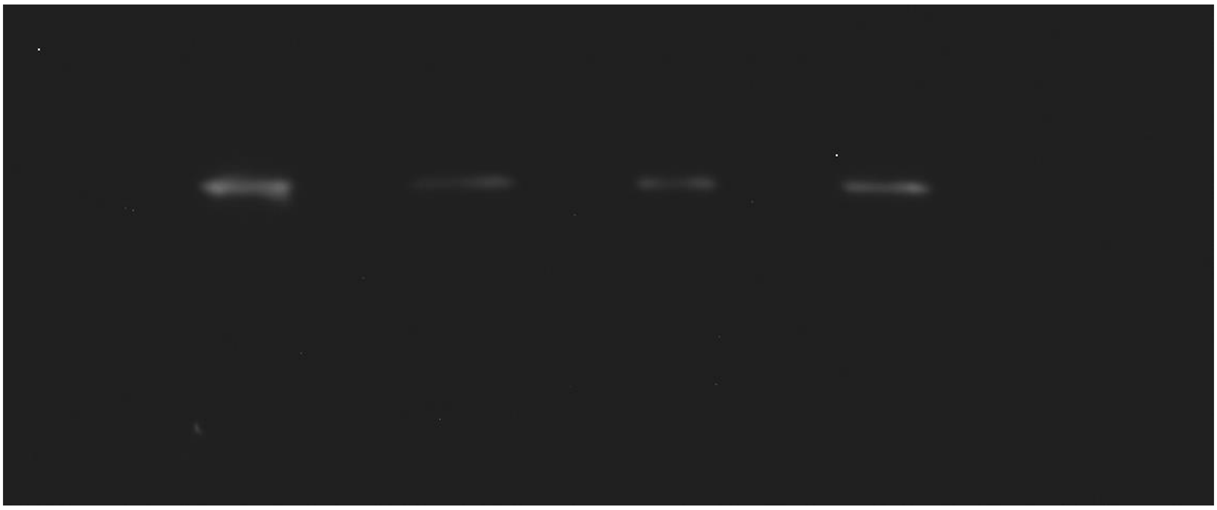

Figure 3D Marker for IκB blot

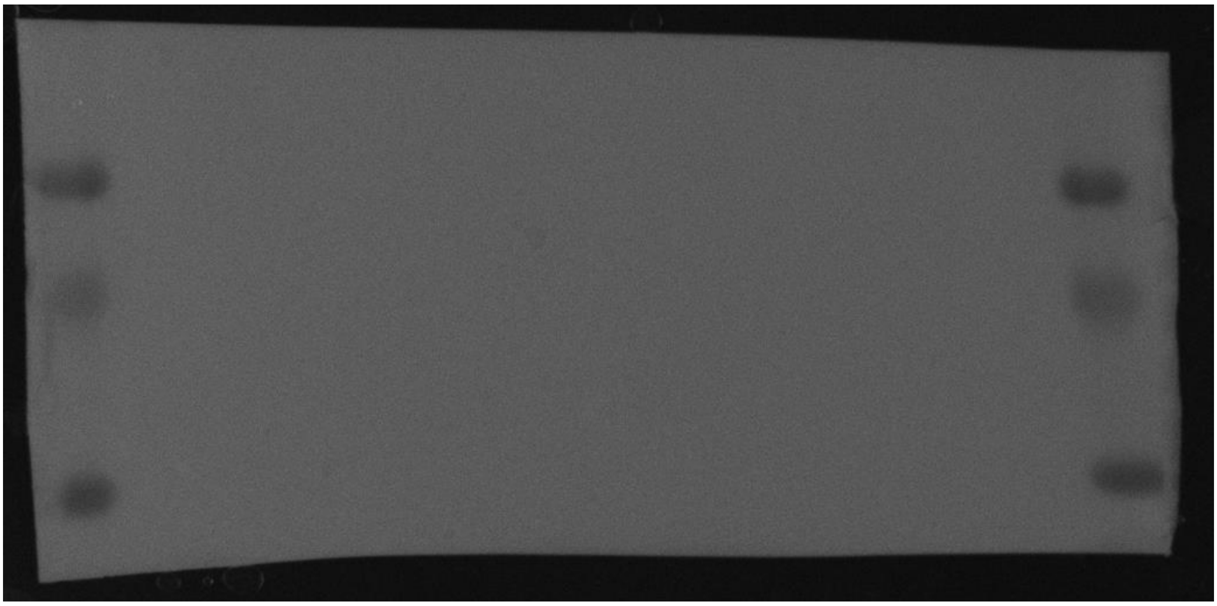

Figure 3D Vinculin exposure time 8 min.

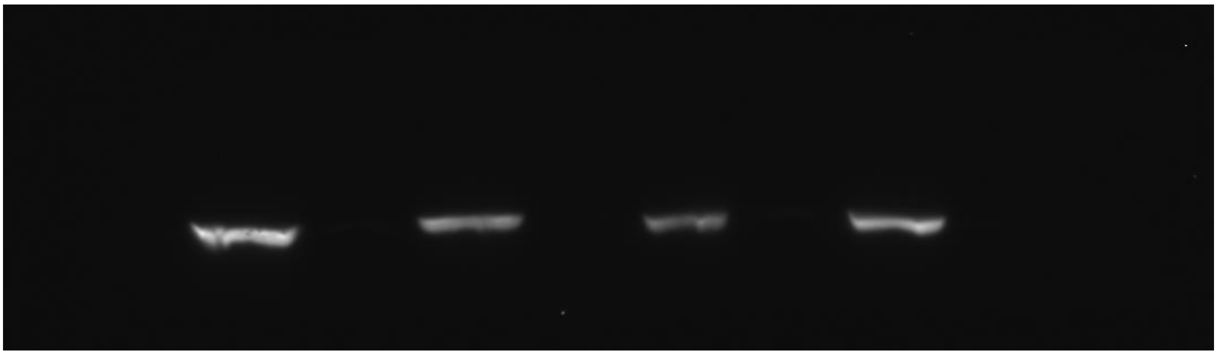

Figure 3D Marker for Vinculin blot

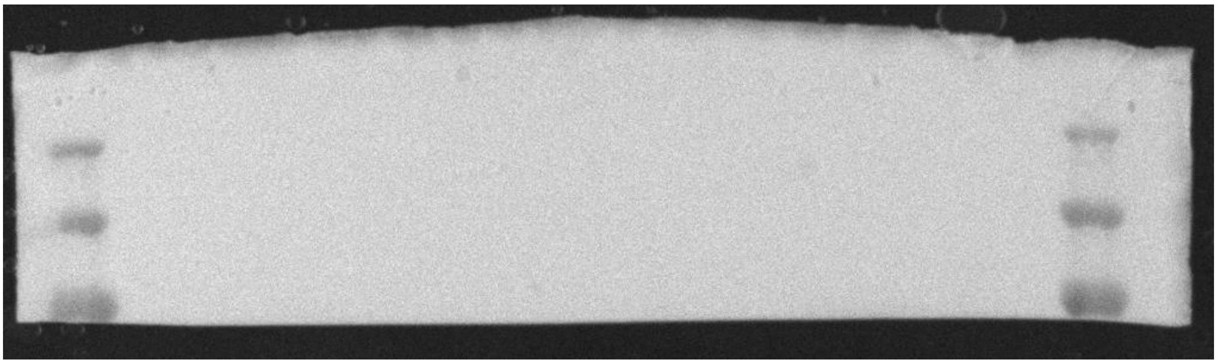

Figure S3 Complete gel

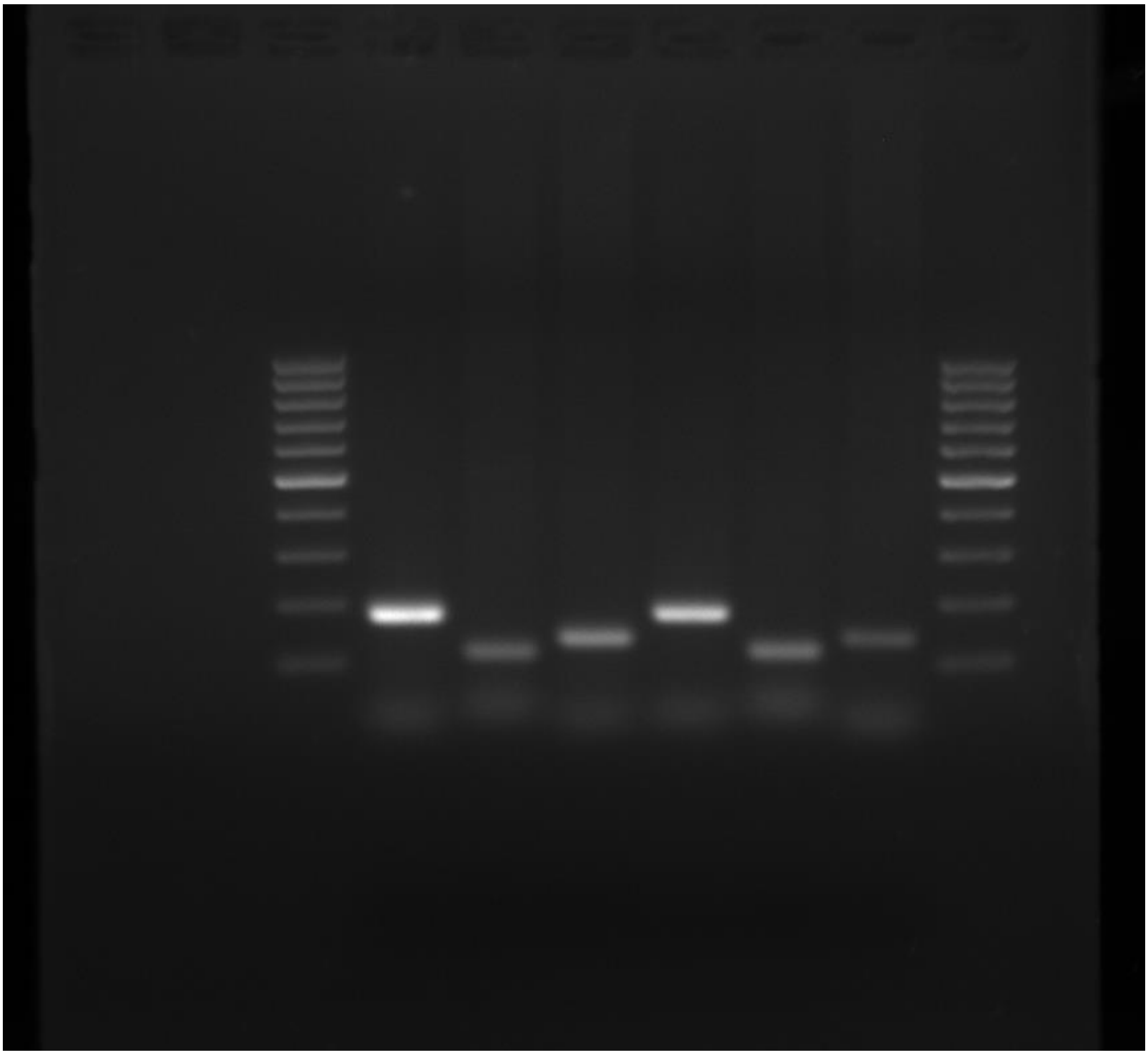

Figure S4 TLR2 exposure time 20 min.

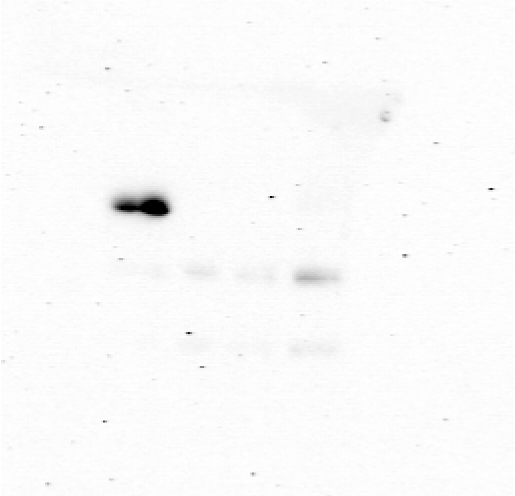

Figure S4 Marker for TLR2 blot

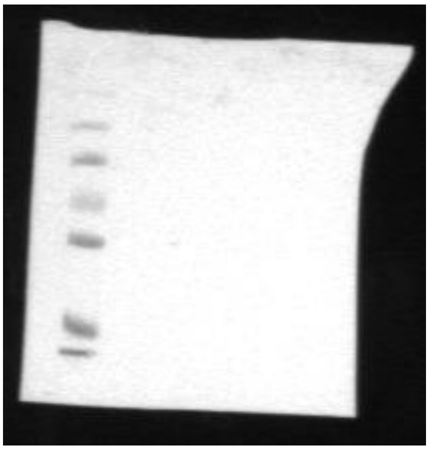

Supplement: Supplementary file 2 — Supplementary Material 2 [file 41598_2026_62091_MOESM2_ESM.pdf]
